# Soil water availability and branch age explain variability in xylem safety of European beech in Central Europe

**DOI:** 10.1007/s00442-022-05124-9

**Published:** 2022-02-25

**Authors:** Greta Weithmann, Roman M. Link, Bat-Enerel Banzragch, Laura Würzberg, Christoph Leuschner, Bernhard Schuldt

**Affiliations:** 1 Plant Ecology, Albrecht Von Haller Institute for Plant Sciences, University of Goettingen, Untere Karspüle 2, 37073 Göttingen, Germany; 2 Present Address: Ecophysiology and Vegetation Ecology, Julius-von-Sachs-Institute of Biological Sciences, University of Würzburg, Julius-von-Sachs-Platz, 97082 Würzburg, Germany; 3 Centre for Biodiversity and Sustainable Land Use (CBL), University of Goettingen, 37075 Göttingen, Germany

**Keywords:** Available soil water capacity, Climatic water balance, Embolism resistance, Hegyi competition index, Hydraulic conductivity, Hydraulic plasticity, Precipitation gradient, Xylem vulnerability curve

## Abstract

Xylem embolism resistance has been identified as a key trait with a causal relation to drought-induced tree mortality, but not much is known about its intra-specific trait variability (ITV) in dependence on environmental variation. We measured xylem safety and efficiency in 300 European beech (F*agus sylvatica* L.) trees across 30 sites in Central Europe, covering a precipitation reduction from 886 to 522 mm year^−1^. A broad range of variables that might affect embolism resistance in mature trees, including climatic and soil water availability, competition, and branch age, were examined. The average *P*_50_ value varied by up to 1 MPa between sites. Neither climatic aridity nor structural variables had a significant influence on *P*_50_. However, *P*_50_ was less negative for trees with a higher soil water storage capacity, and positively related to branch age, while specific conductivity (*K*_s_) was not significantly associated with either of these variables. The greatest part of the ITV for xylem safety and efficiency was attributed to random variability within populations. We conclude that the influence of site water availability on *P*_50_ and *K*_s_ is low in European beech, and that the high degree of within-population variability for *P*_50_, partly due to variation in branch age, hampers the identification of a clear environmental signal.

**Supplementary Information:**

The online version contains supplementary material available at 10.1007/s00442-022-05124-9.

## Introduction

Drought-induced tree mortality has been documented worldwide (Allen et al. 2010, 2015; Hartmann et al. 2018), including European temperate forests (Braun et al. 2020; Schuldt et al. 2020). The capability of different tree species to survive extreme drought intensities varies, but reliable trait-based predictions of future climate warming-related vitality reductions and forest community changes do rarely exist due to insufficient knowledge of plant trait variability (cf. Berzaghi et al. 2020). Because natural selection acts on heritable variation, knowledge of the degree of within-population trait variability is essential to evaluate the capacity of a species to cope with climate change (Nicotra et al. 2010). However, although growing in numbers, to date, relatively few field studies have quantified the variability in plant hydraulic traits across a species’ range (e.g., Martínez-Vilalta et al. 2009; Schuldt et al. 2016; Stojnic et al. 2018; Rosas et al. 2019; Fajardo et al. 2020; Fuchs et al. 2021).

In Central Europe, European beech (*Fagus sylvatica* L.) is the dominant species of natural forest vegetation and occurs under widely different precipitation regimes (Leuschner and Ellenberg 2017). For colonizing such a wide spectrum of habitats, a high degree of intra-specific trait variability is a likely prerequisite. In the recent past, however, mass mortality of European beech after extreme drought events has been reported not only from locations at the range edge (e.g., Lakatos and Molnár 2009) but also from the centre of its distribution range (Braun et al. 2020; Leuschner 2020; Schuldt et al. 2020). This matches earlier findings from physiological studies on the species’ drought sensitivity showing low water potentials, reduced nitrogen uptake, and a high water use efficiency under dry conditions (Rennenberg et al. 2004; Geßler et al. 2006) and dendroecological evidence of long-term growth declines in various Central European beech forest regions (review in Leuschner 2020). It therefore remains questionable whether the intra-specific variability in plant hydraulic traits observed across marginal populations (Stojnic et al. 2018) is likewise present in the centre of the species’ distribution range.

While the processes causing drought-induced tree mortality are complex, one key mechanism involved in tree mortality upon drought is the partial or complete loss of xylem functionality due to embolism formation (Adams et al. 2017; Choat et al. 2018; Brodribb et al. 2020). Even though a number of co-occurring risk factors have been identified, plant hydraulic traits, foremost xylem embolism resistance, have been related to the survival success of trees after severe droughts in various forest regions (Rowland et al. 2015; Anderegg et al. 2016; Adams et al. 2017; Tai et al. 2017; Correia et al. 2019; Hajek et al. 2020; Li et al. 2020; Powers et al. 2020). Upon drought stress, the tension in the water conducting conduits increases and may cause embolism in the xylem and eventually lead to the collapse of the hydraulic system (Tyree and Sperry 1989). Hence, maintaining the continuity of the water column in the conduit system is essential for vascular plants (Hacke et al. 2017). Species comparisons have revealed that embolism resistance generally increases with climatic aridity (Maherali et al. 2004; Choat et al. 2007; Larter et al. 2017; Li et al. 2018; Skelton et al. 2021). For temperate broad-leaved species and at the intra-specific level, however, results are mixed (e.g., Schuldt et al. 2016; Rosas et al. 2019; Fuchs et al. 2021).

The capacity of European beech to form productive forests in various regions of Western, Central, and Eastern Europe with oceanic-to-continental climates suggests considerable phenotypic plasticity, i.e., the ability to acclimate to different environmental conditions, or high genotypic variation in its distribution range (Bolte et al. 2007; Meier and Leuschner 2008; Bresson et al. 2011). Because natural selection always involves the inheritance of genetic information, however, it is a logical consequence to address the amplitude of phenotypic-plastic responses in ecological studies (Olson 2019). Population genetic studies have shown that within-population genetic variance is usually larger than genetic differences between European beech populations, at least in the centre of its distribution range (Buiteveld et al. 2007; Carsjens et al. 2014). In correspondence, earlier studies revealed a high intra-population variability of embolism resistance in European beech stands (Herbette et al. 2010; Wortemann et al. 2011; Aranda et al. 2015; Hajek et al. 2016). Significant differences in embolism resistance between populations, on the other hand, were only observed for marginal populations (Stojnic et al. 2018), and in one study comparing stands of similar age and structure on similar soils in Germany (Schuldt et al. 2016), which differed only little in their genetic structure (Carsjens et al. 2014). This leads to the hypothesis that differences in embolism resistance in European beech are largely caused by the selective force of the local environment that leads to adaptive modifications in the traits of the individuals (Schuldt et al. 2016; Stojnic et al. 2018), but it remains speculative why others did not observe a clear climatic signal (Herbette et al. 2010; Wortemann et al. 2011; Rosas et al. 2019). One reason for the contradicting results might be the selection of different explanatory variables. Most field studies along climatic gradients did neither include soil texture and soil water storage capacity, nor branch age in the analysis. This is surprising, as all of these variables have been found to influence xylem hydraulic properties, at least in certain species (Schuldt et al. 2016; Waite et al. 2019).

Soil physical properties are important as determinants of soil water storage capacity that directly impact the supply of water and nutrients to the plant. Across all climatic zones, soil depth and texture are important variables in attempts to predict drought-induced tree mortality (O’Brien et al. 2017). This has been confirmed, for example, in Norway spruce, where the risk of mortality seems to depend on soil conditions (Rehschuh et al. 2017), and the inclusion of soil characteristics in models of tree and shrub mortality improved their predictions (Tai et al. 2017; Renne et al. 2019). Furthermore, there is evidence that xylem safety closely correlates with local soil water conditions (Beikircher and Mayr 2009; Awad et al. 2010).

Water availability depends not only on climatic and edaphic conditions, but also on competition between neighbouring trees, which therefore may affect the mortality of trees in forest stands (Das et al. 2011; Ruiz-Benito et al. 2013; Young et al. 2017; Hajek et al. 2020). In European beech*,* intra-specific competition between neighbouring trees was found to influence the drought response, leading for example to reduced stomatal conductance when exposed to competition at dry sites (Baudis et al. 2014). Furthermore, it has been hypothesized that differences in belowground competition might mask the effect of environmental drivers of xylem safety (Fuchs et al. 2021).

On the individual level, tree height (or flow path length) influences xylem architecture through an increasing conduit size towards the stem base (Anfodillo et al. 2013; Olson et al. 2014, 2021; Fajardo et al. 2020). With increasing height and flow path length, the water potential gradient necessary to maintain a given flow rate increases due to the influence of both gravitational force and friction (Koch et al. 2004; Woodruff et al. 2004; Ishii et al. 2008; Ambrose et al. 2016). Consequently, it would be advantageous for the tree to increase embolism resistance with increasing height. Indeed, studies comparing branches at different heights within a tree indicate that xylem safety is positively associated with height (Burgess et al. 2006; Woodruff et al. 2008). This contradicts findings, foremost from the tropics, that taller trees are less embolism resistant than smaller ones, both at the intra- and inter-specific level (Rowland et al. 2015; Olson et al. 2018; Liu et al. 2019; but see Bittencourt et al. 2020). Most likely, the latter can be attributed to a higher transpirational demand of taller trees exposed to a drier atmosphere in environments where water availability is commonly not limiting (cf. Olson et al. 2020).

Another important factor potentially associated with embolism resistance is hydraulic efficiency, which is typically assumed to scale positively with conduit size (Tyree et al. 1994; Gleason et al. 2016). Generally, narrow xylem conduits are considered to be safer than wider ones, leading to the paradigm that there is a safety-efficiency trade-off (Hacke et al. 2017). In branches of European beech, however, no trade-off between xylem safety and hydraulic efficiency was observed (Cochard et al. 1999; Hajek et al. 2016). Yet, branch age was found to correlate positively with the xylem pressure at 50% loss of hydraulic conductance (*P*_50_), i.e., younger branches were found to be more resistant than older branches of comparable diameter (Schuldt et al. 2016). Thus, neither the effects of soil hydrology nor those of stand structure on embolism resistance are well understood.

In the present study, we measured branch xylem safety and hydraulic efficiency in 300 mature European beech trees from 30 stands across a climatic gradient from oceanic to sub-continental, mirrored in a reduction in mean annual precipitation (MAP) by 364 mm year^−1^. We hypothesized that embolism resistance of European beech populations is influenced by (1) site water availability, as determined jointly by climatic and edaphic factors, (2) competition between neighbouring trees, and (3) branch age. From the existing reports, we expected a higher embolism resistance for drier sites, competitively inferior trees, and for younger branches of a given size.

## Materials and methods

### Study sites and climatic conditions

**Fig. 1:**
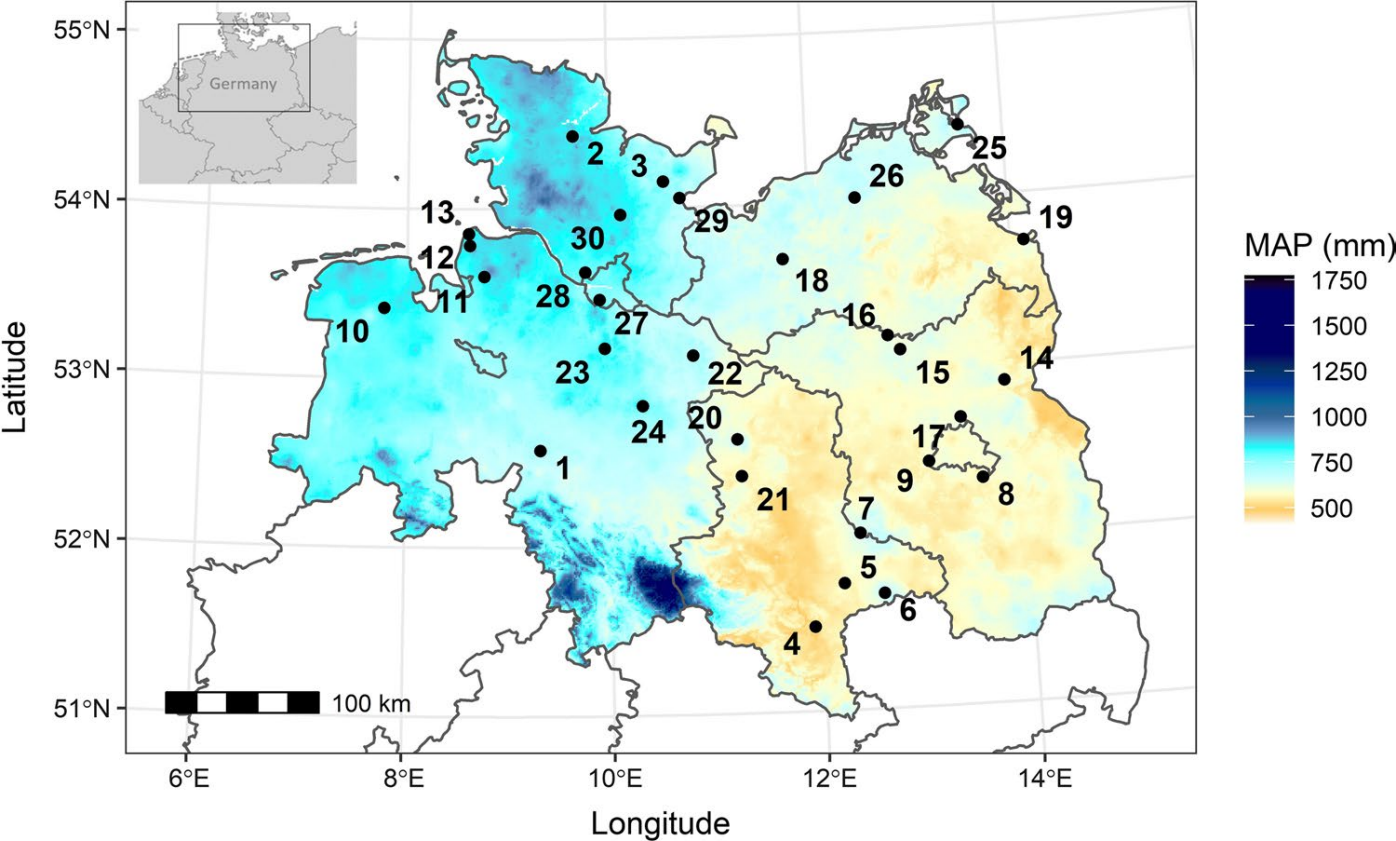
Map of the northern part of Germany with federal states and the location of the 30 investigated beech stands. Colours indicate mean annual precipitation (MAP, 1991–2018, data provided by DWD). For site codes and more physiographic information, see Table 1 and Table S1

The study was carried out at 30 mature European beech (*Fagus sylvatica* L.) stands in the centre of the species distribution range across a gradient from a cool-temperate oceanic to sub-continental climate in the lowlands of northern Germany between the North Sea coast and the Polish border (Fig. 1). The stands were monospecific and grew at elevations of 19–159 m a.s.l. on predominantly nutrient-poor sandy pleistocene soils with a soil depth > 120 cm. Mean annual precipitation (MAP) decreased from West to East from 886 to 522 mm year^−1^, while mean annual temperature (MAT) increased from 9.0 to 10.0 °C. All stands have a cohort-like age structure and are managed for silvicultural purposes, but the last thinning occurred at least 7 years ago, and canopy closure was > 90% in all cases.

The values for monthly precipitation, air temperature, and potential evapotranspiration were extracted for the period from 1991 to 2018 from the Climate Data Centre (CDC) of the German Weather Service (DWD, Deutscher Wetterdienst, Offenbach, https://opendata.dwd.de/, accessed 2019-11-14) using the R package rdwd v. 1.2.0 (Boessenkool 2019). The climate variables for the 30 sites were calculated from extrapolated 1 km-gridded data. The extracted precipitation and potential evapotranspiration data were then used to calculate the monthly cumulative water balance (CWB, mm) as the difference between precipitation and potential evapotranspiration. As mean early growing-season precipitation (April–June, MSP) and mean growing-season precipitation (MGSP) were highly correlated with mean annual precipitation (MAP; Fig. S1), we decided to include the full-year means of the climatic variables in our analyses.

### Soil characteristics

To estimate plant-available soil water capacity (AWC, mm) at the 30 sites and to characterize the soil chemical regime, soil samples were taken from three different depth layers (0–10 cm, 10–30 cm and 30–60 cm) in each two soil pits dug in every stand. We determined the soil organic carbon-to-nitrogen ratio (C_org_/N_t_), total phosphorus content, and soil pH. Subsequently, the stone content (> 2 mm) was determined by sieving a soil volume of 45,000 cm^3^ that was excavated in one pit per stand. Soil bulk density and soil particle-size distribution, i.e., the relative proportion of sand- (63–2000 µm), silt- (2.0–63 µm), and clay-sized (< 2.0 µm) particles, were also determined for three soil depths per stand within one soil pit. The sand fraction was determined by sieving, while the silt and clay fractions were measured by differential sedimentation (PARIO Soil Particle Analyser, METER Group AG, Munich, Germany) (Table S1). From particle-size distribution and soil bulk density, soil hydraulic properties [the van Genuchten (1980) parameters] were derived by deploying pedotransfer functions according to Schaap et al. (2001), using the module “Rosetta light”, implemented in the Software RETC (version 6.02, van Genuchten et al. 1991). The volumetric water contents at permanent wilting point and field capacity were then retrieved from the estimated retention curves using the conventional water potentials of − 1.5 MPa (pF 4.2) and − 60 hPa (pF 1.8). Soil AWC, defined as the water content between field capacity and permanent wilting point, was then calculated for the three depth layers to 60 cm and extrapolated to a standard depth of 100 cm, assuming a homogenous soil particle composition in the 30–100 cm layer. None of the sites had shallow soils, and soil depth exceeded 100 cm in all cases.

### Tree dimensions, competition intensity, and collection of branch samples

Within each stand, ten mature European beech trees of similar size and canopy position were selected. Tree age ranged from 62 to 164 years, diameter at breast height (DBH) from 36 to 58 cm, and tree height from 21 to 34 m across the 30 sites (Table 1). All sample trees were dominant individuals in the upper canopy layer. For estimating competition intensity in the direct neighbourhood of our sample trees, we calculated the Hegyi competition index (CI; Hegyi 1974) for each tree *i* from the distance and height of the three nearest neighbouring trees *j* as

**Table 1:** Stand characteristics of the 30 investigated European beech (*Fagus sylvatica*) forests

Site	Name	Elevation	MAP	MSP	CWB	MAT	AWC	Tree age	DBH	Height	CI
1	Grinderwald	85	720.7	162.1	10.19	9.9	301.0	88.5 ± 4.7	44.9 ± 1.0	26.6 ± 0.6	0.49 ± 0.04
2	Brekendorf	99	878.3	179.0	25.56	9.0	226.8	110.5 ± 1.4	45.6 ± 2.3	27.1 ± 0.5	0.59 ± 0.08
3	Malente	77	752.8	165.4	14.93	9.1	252.5	108.0 ± 3.7	58.2 ± 2.8	30.0 ± 0.4	0.44 ± 0.05
4	Halle	124	522.0	137.6	− 10.82	10.0	286.4	87.0 ± 1.7	45.1 ± 0.5	25.2 ± 0.7	0.49 ± 0.03
5	Mosigkauer Heide	80	565.8	142.9	− 7.31	10.0	177.8	93.6 ± 0.8	44.5 ± 1.2	28.1 ± 0.5	0.53 ± 0.05
6	Dübener Heide	159	673.3	157.4	3.12	9.5	90.7	86.2 ± 1.9	42.0 ± 2.2	26.8 ± 0.5	0.64 ± 0.08
7	Medewitz	142	653.1	154.8	1.87	9.5	126.0	95.3 ± 2.1	47.9 ± 2.5	28.8 ± 0.5	0.54 ± 0.04
8	Zeuthen	47	575.7	145.8	− 7.32	9.7	169.4	80.5 ± 1.1	40.2 ± 0.8	28.1 ± 0.6	0.60 ± 0.06
9	Potsdam	45	582.3	144.3	− 6.14	9.9	197.6	94.5 ± 2.3	35.7 ± 1.1	21.1 ± 0.3	0.58 ± 0.08
10	Wiesmoor	19	819.7	172.5	19.01	9.7	57.7	84.3 ± 2.4	43.2 ± 1.6	27.1 ± 0.4	0.51 ± 0.03
11	Drangstedt	30	858.4	181.3	22.59	9.6	203.0	98.9 ± 2.2	53.7 ± 2.7	34.4 ± 0.8	0.44 ± 0.04
12	Nordholz	33	886.2	185.7	25.23	9.6	103.7	84.7 ± 1.8	44.5 ± 1.4	30.6 ± 0.3	0.51 ± 0.09
13	Sahlenburg	23	849.0	177.7	22.23	9.7	75.7	91.9 ± 1.3	42.3 ± 1.9	26.1 ± 0.6	0.57 ± 0.06
14	Chorin	64	571.4	142.1	− 6.02	9.6	118.6	91.4 ± 2.8	49.6 ± 2.4	30.9 ± 0.6	0.46 ± 0.04
15	Warenthin	81	610.7	149.5	− 0.60	9.2	146.6	139.9 ± 6.2	46.2 ± 1.6	29.2 ± 0.5	0.44 ± 0.05
16	Zempow	109	627.8	153.0	2.19	9.0	188.7	102.3 ± 4.4	40.5 ± 1.4	28.8 ± 0.4	0.41 ± 0.03
17	Summt	58	601.9	145.1	− 3.49	9.8	43.4	89.9 ± 2.1	45.1 ± 1.5	27.5 ± 0.4	0.52 ± 0.05
18	Kaarzer Holz	70	655.3	156.2	5.64	9.2	78.9	94.7 ± 4.7	45.4 ± 1.6	28.3 ± 0.7	0.40 ± 0.03
19	Eggesiner Forst	32	584.5	149.4	− 1.71	9.1	130.4	112.3 ± 5.3	44.0 ± 1.5	27.2 ± 0.5	0.39 ± 0.07
20	Klötze	116	648.4	153.0	2.74	9.4	260.2	131.4 ± 4.4	47.9 ± 1.5	33.9 ± 1.0	0.61 ± 0.06
21	Calvörde	87	572.8	139.9	− 4.65	9.7	159.5	106.8 ± 3.8	42.0 ± 1.1	26.5 ± 0.4	0.59 ± 0.06
22	Göhrde	94	718.7	164.2	10.48	9.2	172.1	164.6 ± 10.2	46.1 ± 2.5	26.7 ± 0.6	0.49 ± 0.03
23	Sellhorn	144	863.2	192.3	24.32	9.0	161.3	121.0 ± 4.9	43.0 ± 1.1	30.1 ± 0.7	0.62 ± 0.04
24	Unterlüß	141	804.7	174.5	17.93	9.1	127.1	110.6 ± 3.4	46.3 ± 1.5	28.9 ± 0.6	0.43 ± 0.04
25	Prora	37	646.3	152.4	5.87	9.1	218.3	122.1 ± 5.7	48.1 ± 1.9	28.0 ± 0.4	0.56 ± 0.05
26	Tessin	49	663.3	158.8	6.40	9.0	166.2	85.1 ± 1.5	43.3 ± 1.4	30.7 ± 0.5	0.62 ± 0.05
27	Haake	72	798.0	179.3	17.45	9.7	88.4	153.7 ± 6.6	47.1 ± 2.0	28.4 ± 0.6	0.59 ± 0.06
28	Klövensteen	34	799.0	175.3	17.48	9.6	158.2	119.0 ± 2.7	46.5 ± 1.5	31.1 ± 0.6	0.46 ± 0.01
29	Haffkrug	51	707.8	157.3	10.81	9.2	256.4	62.4 ± 1.4	42.2 ± 1.9	27.9 ± 0.6	0.48 ± 0.05
30	Heidmühlen	68	851.5	179.8	22.69	9.2	164.2	138.2 ± 5.4	44.3 ± 1.3	27.5 ± 0.5	0.73 ± 0.09

Given are site number (see Fig. 1), location name, elevation (m a.s.l.), mean annual precipitation (MAP, mm year^−1^), mean early growing-season precipitation (April–June; MSP, mm), mean climatic water balance (CWB, mm month^−1^), and mean annual temperature (MAT, °C) for the period 1991–2018, plant-available water capacity (AWC, mm), tree age, diameter at breast height (DBH, cm), tree height (Height, m), and the Hegyi competition index (CI). Climate data were retrieved from the Climate Data Centre of the German Weather Service (DWD, Offenbach). For data on tree level, means per site ± SE are given

$${\mathrm{CI}}_{i}= \sum_{j=1}^{n}\frac{{d}_{j}/{d}_{i}}{{\mathrm{Dist}}_{ij}},$$

where *d*_*i*_ is the diameter at breast height of the sampled tree *i* (cm), *d*_*j*_ the diameter at breast height of the competitor *j* (cm), Dist_*ij*_ the distance between target tree and competitor (m), and *n* the number of directly neighbouring trees taken into account (= 3).Long branch samples were collected in the summers 2018 and 2019 (June–August) from the uppermost sun-exposed crown by professional tree climbers. On the ground, three lateral branches with maximum 1 m length were cut; one of them was selected for hydraulic measurements later. The distance to the branch tip from the basipetal ends of the branch samples was estimated from diameter–length measurements of 85 upper canopy beech branches (Hajek et al. 2015) and ranged from 37 to 91 cm with an average of 60.08 ± 0.55 cm (Weithmann et al., in review). The air-cut ends of the branches were immediately transferred into a water-filled bucket and recut under water to release xylem tension. After ~ 20 min, small side-branches were shortened to a length of ca. 1 cm, branch segments wrapped in wet towels, sealed in plastic bags, and stored at a temperature of 7 °C until further processing within 2 weeks.

### Hydraulic measurements

In the laboratory, one sample per tree was selected for the hydraulic measurements. The branches of 8.97 ± 0.06 mm (mean ± SE) basipetal diameter were shortened to a length of 34.16 ± 0.11 cm (mean ± SE), lateral ﻿branches cut off and sealed with quick-drying instant glue (Loctite 431, Henkel, Düsseldorf, Germany), the bark removed at the basipetal end, and segments connected to a Xyl’em embolism meter (Bronkhorst France, Montigny les Cormeilles, France). After measuring the initial hydraulic conductivity (*K*_h_, kg m MPa^−1^ s^−1^) at low pressure (6 kPa), samples were flushed up to four times at high pressure (120 kPa) for 10 min with filtered, degassed, and demineralized water containing 10 mM KCl and 1 mM CaCl_2_ until no further increase in *K*_h_ was observed. Specific conductivity (*K*_s_, kg m^−1^ MPa^−1^ s^−1^) was calculated by dividing maximum *K*_h_ obtained from the Xyl’em measurements after the flushing procedure by the xylem cross-sectional area without pith and bark (*A*_xylem_, mm^2^), which was estimated from the branch cross-sectional area (*A*_cross_, mm^2^) as ${A}_{\mathrm{xylem}}= -3.715 + 0.770\times {A}_{\mathrm{cross}}$ (Schuldt et al. 2016). Subsequently, the same branch segments were shortened to 27.5 cm, the bark was removed at both ends, and xylem vulnerability curves were constructed with the flow-centrifuge technique (Cavitron; Cochard et al. 2005). In European beech, an average maximum vessel length of 19.3 ± 2.6 cm has been reported (Lübbe et al. 2021), which makes this species suited for flow-centrifuge measurements with a 30 cm-rotor. Segments were inserted into a custom-made honey-comb rotor attached to a Sorvall RC-5C centrifuge (Thermo Fisher Scientific, Waltham, MA, USA). While spinning, conductivity was calculated continuously by the software CaviSoft (version 4.0, University of Bordeaux, France). Measurements started at a pressure of − 0.37 MPa, which was raised stepwise until 90% loss of hydraulic conductivity was reached.

Analogous to Ogle et al. (2009), we estimated the parameters of the vulnerability curves based on *K*_s_ instead of converting measured conductivities into percent loss of conductivity. To do so, we reformulated the sigmoidal model of Pammenter and Vander Willigen (1998) to obtain the following equation for the expected value of *K*_s_ for observation *i* of sample *j*:

$${K}_{{s}_{ij}}= {K}_{{\mathrm{max}}_{j}}\left(1- \frac{1}{1+\mathrm{exp}\left({s}_{j}\,\left({{\Psi }_{i}-P}_{{50}_{j}}\right)\right)}\right),$$

where *Ψ*_i_ is the pressure induced by the rotation of the rotor (corresponding to xylem water potential), *K*_max_ the estimated maximum conductivity, *P*_50_ the water potential at 50% loss of conductance, and *s* the slope of the regression line on the logit scale. The xylem pressures at 12% and 88% loss of conductance (*P*_12_ and *P*_88_, respectively) were calculated by inserting the desired quantiles of loss of conductance into the model equation of Pammenter and Vander Willigen (1998) and solving for the corresponding water potential.

To assess a potential effect of the flushing procedure on results of the flow-centrifuge measurements, *P*_50_ values of non-flushed branches of 13 trees were compared to flushed branches of the same trees. 20 branches were harvested from site number 28 in August 2018 at the end of the sampling campaign, and 6 were collected from three other sites end of July in 2018.

### Branch age

From the basipetal and acropetal end of the branch samples used for hydraulic measurements, semi-thin transverse sections were cut with a sliding microtome (G.S.L.1; Schenkung Dapples, Zurich, Switzerland) and growth rings counted at ×100 magnification under a stereo-microscope (SteREOV20; Carl Zeiss MicroImaging GmbH, Göttingen, Germany) for estimating the age of the branch at both ends. For all subsequent analyses, we estimated the mean of the basipetal and acropetal branch age.

### Statistical analyses

All statistical analyses were performed with the R version 3.6.2 (R. Core Team 2019) in the framework of the tidyverse package (Wickham et al. 2019). Five separate linear mixed-effects models were fitted with the R package lme4 v. 1.1-23 (Bates et al. 2015) using Restricted Maximum Likelihood with *P*_50_, *P*_12_, *P*_88_, slope, and *K*_s_ as responses, fixed effects for CWB, AWC, CI, tree height, and branch age, and random intercepts for sites.

As precipitation averages over different timescales were highly correlated and as mentioned above, spring and growing-season precipitation were tightly associated with full-year precipitation (Fig. S1), and data for potential evapotranspiration, which are needed for calculating CWB, are only provided from 1991 onwards; mean annual CWB of the period 1991–2018 was included in the models as the sole climate variable. As temperature directly influences potential evapotranspiration, it enters the model through CWB.

Data on branch age and slope of the vulnerability curves were log-transformed, and all numeric predictor variables were scaled by their standard deviations and centred around zero. Inference was based on Wald *t* tests with Satterthwaite’s approximation to the degrees of freedom using R package lmerTest v. 3.1-2 (Kuznetsova et al. 2017). The marginal and conditional *R*^2^ (Nakagawa et al. 2017) were computed based on R package MuMIn v. 1.43.17 (Bartón 2020).

To illustrate pairwise linear associations between variables, Pearson correlation analyses were carried out using the R package corrmorant v. 0.0.0.9007 (Link 2020). No problematic level of multi-collinearity was detected in the explanatory variables of the model, with all pairwise correlations being well below 0.7 (Figure S3; cf. Dormann et al. 2013).

To exclude a possible relationship between the mean and total leaf area or tree height and branch age, linear regression analyses were conducted using R function lm(). All leaves distal to the basal end of the segment used for hydraulic measurements were removed, a sub-sample was scanned, and the whole leaf area supported by the branch was calculated from the dry mass and area of the scanned leaves (for details, see Weithmann et al. 2022).

## Results

### Embolism resistance and hydraulic conductivity across trees and sites

**Fig. 2:**
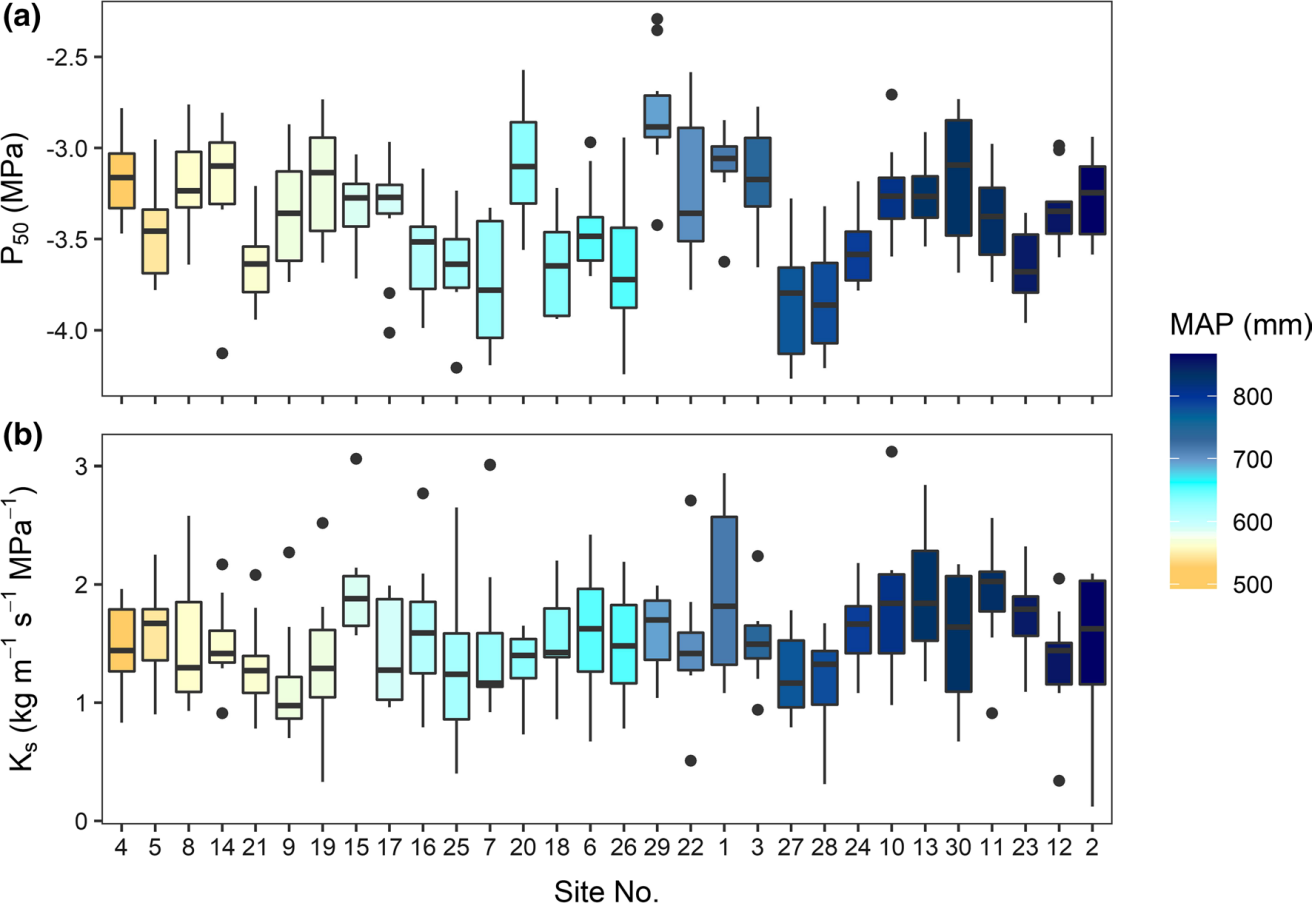
Box plots visualizing **a** *P*_50_ values and **b** specific conductivity (*K*_s_) of branch segments of European beech in the 30 stands (10 trees per stand). Colours indicate mean annual precipitation (MAP, 1991–2018, data provided by DWD) of the different sites (see Fig. 1)

The estimated *P*_50_ values of the branch xylem varied markedly between the different sites, and also between trees within the same site (Fig. 2a). The average *P*_50_ of the 300 studied European beech branches was − 3.38 ± 0.02 MPa (mean ± SE), with site means ranging from − 3.84 ± 0.10 MPa to − 2.82 ± 0.09 MPa (Table S2). The maximal difference between *P*_50_ values among the ten trees per stand was 1.32 MPa. Mean *P*_12_ of the different sites ranged from − 3.03 ± 0.09 MPa to − 1.85 ± 0.09 MPa, and mean *P*_88_ from − 4.75 ± 0.09 MPa to − 3.69 ± 0.07 MPa, respectively. Specific conductivity (*K*_s_) of the branch xylem ranged from 0.12 to 3.12 kg m^−1^ MPa^−1^ s^−1^ with site means varying between 1.14 ± 0.15 kg m^−1^ MPa^−1^ s^−1^ and 1.95 ± 0.14 kg m^−1^ MPa^−1^ s^−1^ (Fig. 2b, Table S2).

### Effect of climatic and soil variables on hydraulic safety and efficiency

**Fig. 3:**
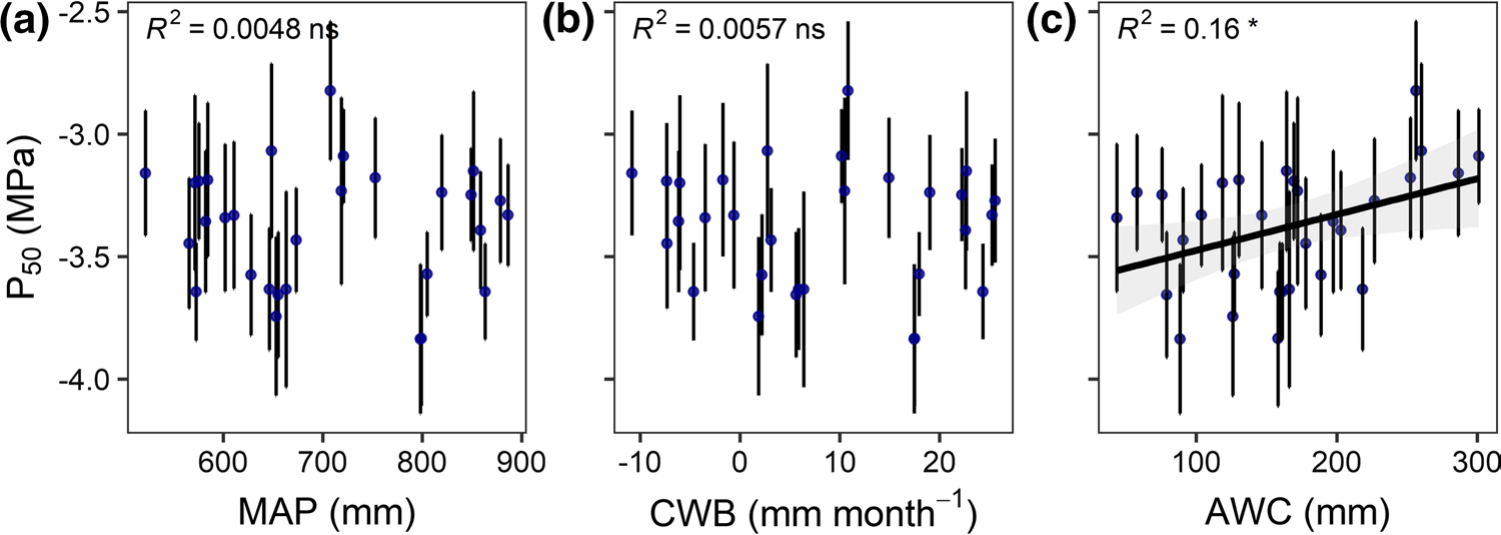
**a** Mean annual precipitation (MAP), **b** climatic water balance (CWB), and **c** available water capacity of the soil (AWC) in relation to the xylem pressure at 50% loss of hydraulic conductance (*P*_50_). Given values are means ± SE per site; asterisks indicate the level of significance and *R*^2^ values the explained variance of the linear regression through the plot averages (*: *p* < 0.05; ns: non-significant relationship). For the significant relationship in **c**, the linear regression line with its 95% confidence intervals is shown

According to the linear mixed-effects (LME) models, the climatic water balance (CWB) as a measure of the climatic water availability was unrelated to any of the variables related to hydraulic safety or efficiency (*P*_12_, *P*_50_, *P*_88_, slope, *K*_s_, Table 2; see Table S3 for standard errors, degrees of freedom and test statistics). The linear regression analyses that were carried out for visualization showed no significant relation of mean annual precipitation (MAP) or CWB and *P*_50_ in agreement with the LME results (Fig. 3a, b). However, we found a significant effect of the plant-available water capacity of the soil (AWC) on *P*_50_ (Table 2), a variable that introduces soil physics into the model and represents the soil water availability. Trees growing on sites with lower AWC developed a more negative *P*_50_ value (Fig. 3c), but both *P*_12_ and *P*_88_ as well as *K*_s_ were unaffected (Table 2). Mean annual temperature (MAT) and soil chemical properties, which were not included as model parameters, were not associated with *P*_50_ or *K*_s_ (Figure S2).

**Table 2:** Results from the linear mixed-effects models with fixed effects for climatic water balance (CWB), plant-available water capacity (AWC), tree height, branch age (log-transformed), and Hegyi competition index (CI), and random intercepts for site on the xylem pressures at 50%, 12%, and 88% loss of conductivity (*P*_50_, *P*_12_, and *P*_88_, respectively), the slope at the water potential at 50% loss of conductance (natural log-transformed), and specific conductivity (*K*_s_; *n* = 298)

	*P*_12_		*P*_50_		*P*_88_		log(slope)		*K*_s_	
	Est	*p*	Est	*p*	Est	*p*	Est	*p*	Est	*p*
Fixed parts										
(Intercept)	− 2.655	** < 0.001**	− 3.381	** < 0.001**	− 4.107	** < 0.001**	1.049	** < 0.001**	1.536	** < 0.001**
CWB	0.031	0.497	0.004	0.929	− 0.023	0.617	− 0.040	0.094	0.026	0.576
AWC	0.089	0.053	0.086	**0.041**	0.083	0.078	0.000	0.941	0.046	0.321
Tree height	0.010	0.773	0.000	0.988	− 0.011	0.697	− 0.024		0.009	0.823
Br. age (log)	0.080	**0.002**	0.088	** < 0.001**	0.097	** < 0.001**	0.015	0.343	− 0.048	0.160
CI	− 0.024	0.340	− 0.011	0.541	0.003	0.893	0.016	0.197	0.022	0.510
Random part										
Site SD	0.205		0.199		0.228		0.118		0.175	
Residual SD	0.381		0.279		0.280		0.258		0.535	
Marginal *R*^2^	0.082		0.128		0.135		0.041		0.016	
Cond. *R*^2^	0.289		0.421		0.480		0.208		0.111	

Est: parameter estimates; *p*: *p*-value for the null hypothesis that a fixed effects parameter is 0 (bold: significant on the 0.05 level)

See Table S3 for standard errors, test statistics, and degrees of freedom

### Influence of branch age, tree height, and competition on embolism resistance

**Fig. 4:**
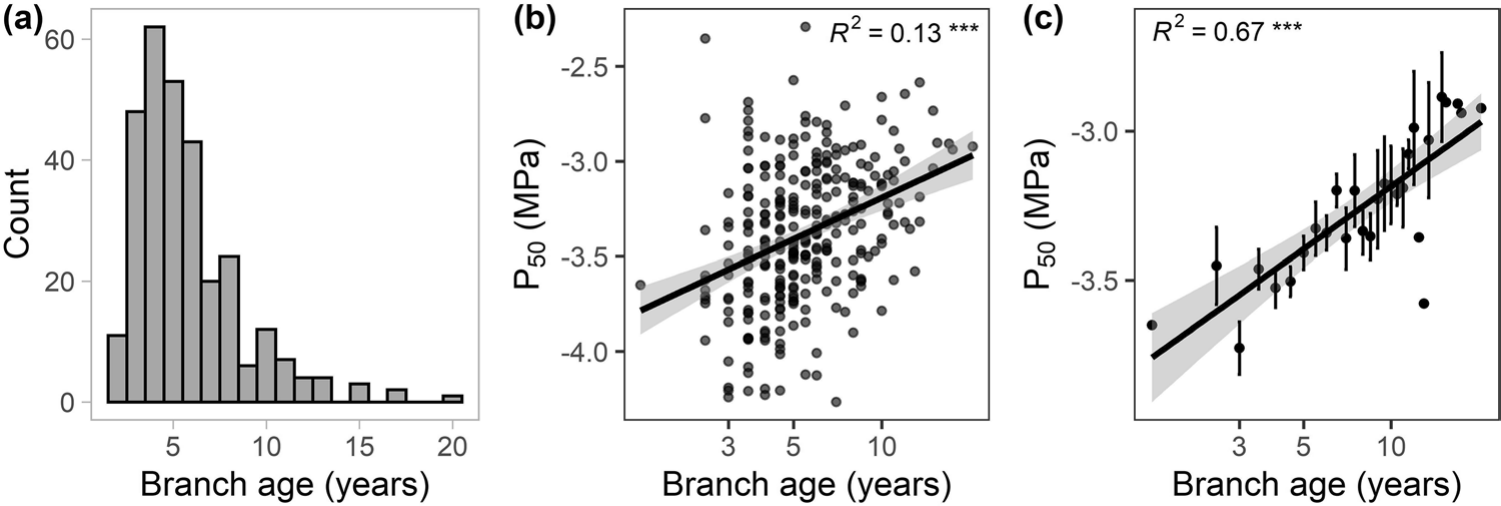
**a** Histogram showing the frequency of branch ages for the 300 samples, as well as tree level linear regression of *P*_50_ against **b** log_10_-transformed mean branch age, and **c** log_10_-transformed mean branch age. Given are the means per given age ± SE, i.e., averaged values per corresponding growth ring, asterisks in **b**, **c** indicate the level of significance and R^2^ values the explained variance of the corresponding linear regression line (***: *p* < 0.001)

**Fig. 5:**
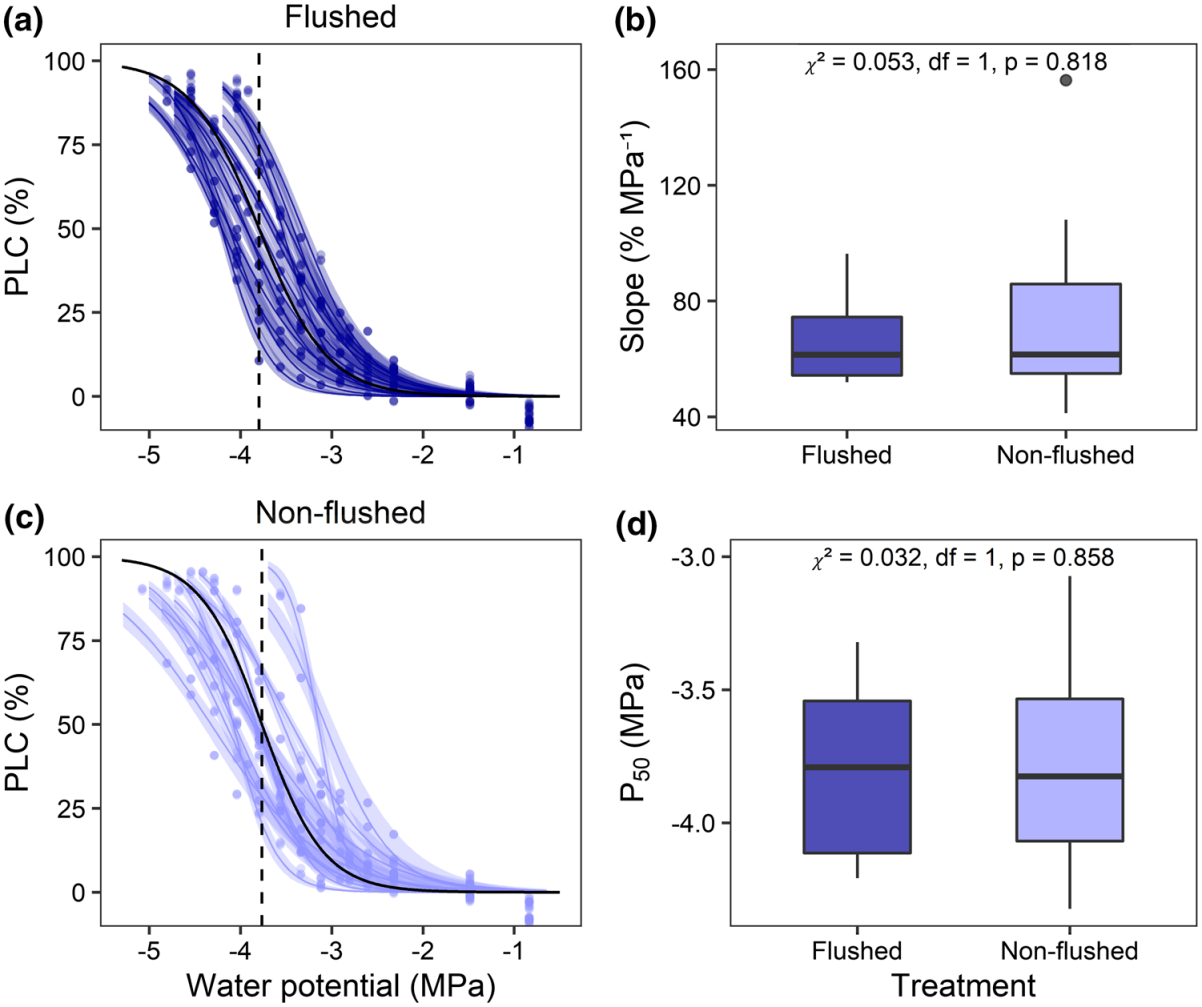
Measured vulnerability curves of **a** flushed vs. **c** non-flushed samples. Shown are observed PLC and the predicted vulnerability curves with their bootstrapped 95% confidence intervals overlaid with the average vulnerability curve (black) and the mean *P*_50_ (black dashed line). Further given are the **b** estimated slope and **d** *P*_50_ for the two treatments with the *p* values and summary statistics from a Kruskal–Wallis test comparing the two treatments

We observed a highly significant effect of branch age on all three measures of embolism resistance (*P*_12_, *P*_50_, and *P*_88_). In contrast, branch age affected neither the slope of the vulnerability curve at the water potential at 50% loss of hydraulic conductance (slope) nor *K*_s_ (Table 2). The mean age of the studied branch samples (averaged between basipetal and acropetal end) ranged from 1.5 to 20.5 years (mean ± SE: 6.0 ± 0.2 years), even though branch diameter was roughly similar (Fig. 4a). Across the gradient, no effect of water availability on branch age was observed (Figure S6), and neither CWB nor AWC had a significant effect on branch age according to a linear mixed-effects model (data not shown). Despite considerable variability within the younger branch age classes, older branches showed higher *P*_50_ values and were less embolism resistant (Fig. 4b, c). In a sub-sample of 13 trees, we could confirm that the observed age effect was not a consequence of the flushing procedure. The constructed vulnerability curves of flushed and non-flushed samples were comparable in their shape (Fig. 5a), and the estimated slope and *P*_50_ value did not differ between both treatments (Fig. 5b). Interestingly, no significant relationship between branch age and branch diameter or the degree of branch tapering (difference between basipetal and acropetal branch diameter) was found (*R*^2^ = 0.006 and 0.004, respectively; data not shown). Branch age at the basipetal end of the segments was significantly related to the age at the acropetal end, and mean branch age was significantly related to the difference between the age of the basipetal and acropetal end (Figure S4). No relationship between branch age and mean or total leaf area of the corresponding branch was observed, and branch age was not related to tree height (Figure S5). Neither the Hegyi competition index (CI) nor tree height showed a significant effect on xylem safety or efficiency (Table 2).

### Variance decomposition

**Fig. 6:**
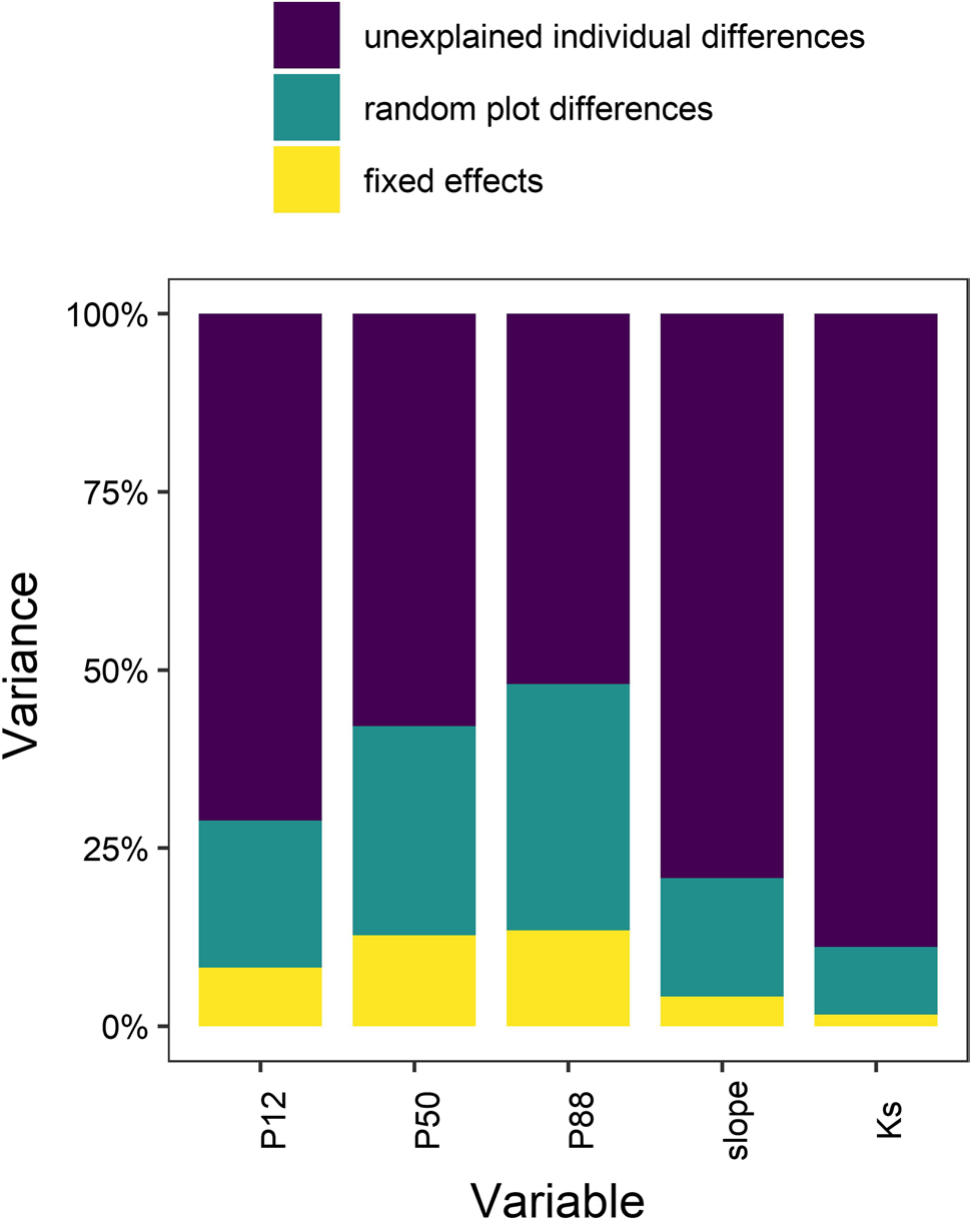
Variance components of the linear mixed-effects models for *P*_12_, *P*_50,_ *P*_88_, slope of the vulnerability curve at *P*_50_, and *K*_s_ with climatic water balance (CWB), plant-available water capacity (AWC), tree height branch age, and Hegyi competition index (CI), as fixed effects, random site effects, and residual variability between individuals (see Table 2)

According to the results of the LME models, the explained variance of the fixed effects (CWB, AWC, CI, tree height, and branch age) was low (~ 13% for *P*_50_, Fig. 6). Differences between sites that could not be attributed to the fixed effects accounted for 29% of the variance in *P*_50_, while 58% of the variance resulted from differences between trees in a stand. The fraction of variance explained by the fixed effects and the random site effect increased from *P*_12_ and *P*_50_ to *P*_88_ from 8 to 13%, and from 21 to 35%, respectively, whereas differences in slope and *K*_s_ were mainly attributable to unexplained differences between individual trees (79% and 89%, respectively).

### No trade-off between safety and efficiency

Pairwise linear correlations of the variables included in the models showed no relationship between *P*_50_ and *K*_s_ at the branch level despite considerable variation in *P*_50_ of 1 MPa (*r* = 0.06, Figure S3).

## Discussion

### Influence of climatic aridity and soil water availability on xylem safety and efficiency

We observed a high degree of intra-specific variability in the xylem pressure at 50% loss of hydraulic conductance (*P*_50_) of sun-canopy branches in mature European beech forests. Across the 30 studied sites in Central Europe, mean *P*_50_ varied from − 2.82 to − 3.84 MPa, which is similar to the range reported in earlier field studies (Herbette et al. 2010; Stojnic et al. 2018). Xylem specific conductivity (*K*_s_) likewise revealed a considerable intra-specific variability, with site means ranging from 1.1 to 2.0 kg m^−1^ MPa^−1^ s^−1^. Despite these pronounced differences in xylem safety and efficiency across sites, observed trait variability could neither be attributed to climatic water availability nor tree and stand structural variables, and the highest proportion of variance was caused by between-tree differences, especially in case of *K*_s_. Although the fixed effects only explained 13% of the total variance in *P*_50_, we observed a significant influence of the plant-available water storage capacity (AWC) of the soil, i.e., soil physical properties that determine soil water availability in rainless periods and the flow resistance experienced by water-absorbing roots in drying soil. Based on earlier research that showed a more embolism-resistant xylem in European beech trees with higher drought exposure (Schuldt et al. 2016; Stojnic et al. 2018), we expected that embolism resistance is influenced by both climatic and edaphic measures of water availability (CWB and AWC). This is also suggested by the studies of Wortemann et al. (2011) and Lübbe et al. (2017), who documented effects of water availability on embolism resistance in European beech, and by other work showing a high degree of hydraulic plasticity in this species (Herbette et al. 2010; 2020; Aranda et al. 2015, 2017; Nguyen et al. 2017; Noyer et al. 2017). To date, however, the existing evidence for an intra-specific increase in xylem safety with increasing climatic aridity in European beech is scarce (e.g., Schuldt et al. 2016; Stojnic et al. 2018). In fact, most field studies on mature trees failed to detect the anticipated adaption of xylem safety to water availability at the intra-specific level in coniferous and broad-leaved (diffuse- or ring-porous) temperate tree species (e.g., Martínez-Vilalta et al. 2009; Martin-StPaul et al. 2013; Lamy et al. 2014; González-Muñoz et al. 2018; Rosas et al. 2019). However, embolism resistance likely depends not only on the precipitation regime, but also on stand structural and edaphic properties. We originally assumed that Schuldt et al. (2016) found a clear climatic effect on *P*_50_ mainly because soil and stand structure were well comparable across their rather local precipitation gradient (cf. Hertel et al. 2013; Müller-Haubold et al. 2013). Because this is usually not the case when larger regions are studied, we additionally included competition index (CI) and soil available water capacity (AWC) as fixed effects in the mixed models. In contrast to Schuldt et al. (2016), the precipitation gradient was steeper in our study (364 mm year^−1^ versus 261 mm year^−1^, respectively) and the *P*_50_ variation across sites was consequently larger (1.02 MPa versus 0.33 MPa, respectively). As we did not find convincing evidence of a significant effect of climatic aridity, expressed through CWB, on embolism resistance, we conclude that soil physical properties that determine water storage capacity must be more important drivers for European beech. This seems plausible in regions where site differences in soil grain size distribution or soil depth cause considerable variance in soil water storage, which may outweigh gradients in summer precipitation. In our study, only beech stands on deep and predominantly sandy soils were included. A greater effect of AWC might be observed when stands on deep and shallow soils are compared, or sites that differ strongly in soil physical properties. However, our results are supportive for the assumption of Carminati and Javaux (2020) that the hydraulic conductivity of the rhizosphere may be the primary driver of stomatal closure during drought. This would require a close coordination between root and rhizosphere conductance and xylem safety of sun-canopy branches.

### Effects of neighbourhood composition and branch age on embolism resistance

Root competition between neighbouring trees may have a large effect on soil water availability, especially in periods with water deficits, as is visible in thinning experiments in forests (McDowell et al. 2006; Moreno and Cubera 2008). We therefore included Hegyi’s competition index as a fixed effect in the models to account for the social status of the individual in the stand, but an effect on embolism resistance did not appear. Tree height was another variable included in the analysis to account for the increase in gravitational force and friction with height in accordance with the hydraulic limitation hypothesis (Ryan et al. 2006), which predicts a vertical decline in *P*_50_ (cf. Burgess et al. 2006; Woodruff et al. 2008; Ambrose et al. 2009). In our study, tree height was largely comparable among the trees of a stand, but mean tree height varied by up to 13.3 m between sites. Yet, tree height was unrelated to *P*_50_, and also unaffected by the climatic and edaphic variability among the sites. A study on the xylem anatomy of the branches used for the hydraulic measurements revealed neither an effect of tree height on vessel diameter nor a relation between vessel diameter and *P*_50_ across our gradient (Weithmann et al., in review). Furthermore, mean leaf area, which is related to vessel diameter at the petiole base (Olson et al. 2021), did not affect embolism resistance or *K*_s_ (results not shown).

As suggested by Schuldt et al. (2016), we further considered branch age as a fixed factor to explain *P*_50_. The authors observed a highly significant positive effect of branch age on *P*_50_ in European beech, i.e., older branches were found to be less resistant. In European beech, an annual length increment in upper canopy branches of 17 cm year^−1^ has been reported (Hajek et al. 2015). This highlights the need for multi-aged branches for the construction of xylem vulnerability curves with the flow-centrifuge technique. In our study, the age of our similar-sized branches (mean ± SE: 8.9 ± 0.1 mm) ranged from 1.5 to 20.5 years (mean ± SE: 6.0 ± 0.2 years) and was the most influential factor affecting embolism resistance (*P*_12_, *P*_50_, and *P*_88_). The large variability in annual growth rate is likely driven by the specific microclimatic conditions at the position of the branch in the uppermost canopy. This assumption is supported by the fact that branch age was unrelated to water availability, tree height, and mean or total leaf area per branch. Nevertheless, branch age significantly affected vessel diameter variation, with smaller vessel diameters found in older branches (Weithmann et al., in review). As older branches had smaller vessels and a lower embolism resistance, we can exclude an indirect effect of vessel diameter variation on *P*_50_. We speculate that the observed age effect results from a “fatigue mechanism”, i.e., physical damage of the pit membranes due to embolisms occurring either after drought (cavitation fatigue) or frost events (frost fatigue) (Hacke et al. 2001; Christensen-Dalsgaard and Tyree 2014; Zhang et al. 2018; Dai et al. 2020). In older branches of similar diameter, a higher portion of conduits has experienced frost or drought events during their lifetime. As a consequence of fatigue, however, we would have expected steeper vulnerability curves for older branches, but no relation of branch age to the slope of the vulnerability curves was observed. To ensure that vulnerability curves included all potentially functional xylem conduits rather than just the portion that was functional at the time of collection, we flushed all samples prior to the construction of vulnerability curves as recommended by Hacke and Sperry (2001). This procedure should increase the comparability of branches harvested under different climatic conditions. However, to date, no widely accepted protocol exists as to whether xylem vulnerability curves should be constructed with flushed or non-flushed samples. To test whether flushing is affecting the results of vulnerability measurements with a flow centrifuge, we investigated the effect of flushing in 80 multi-aged branches of four temperate diffuse-porous tree species. Although flushed samples had a significantly higher hydraulic conductivity, vulnerability curve parameters were unaffected by this treatment (Weithmann et al., in review). Similarly, we could not detect an effect of the flushing procedure on the estimated slope of the curve or the *P*_50_ value in 13 trees studied at one of our sites (Fig. 5). The flushing effect is hence unlikely to be the primary cause of the highly significant influence of branch age on embolism resistance. Rather, novel visualization techniques of in-situ embolism formation indicate that vessels in older annual rings embolize first during desiccation (Fukuda et al. 2015; Knipfer et al. 2019; Lemaire et al. 2020; Meixner et al. 2020). Because all vessels were hydraulically functional in these studies, it remains unclear why older vessels are more vulnerable than younger ones. Possible explanations include frost fatigue or the potential coating of pit membranes by xylem sap surfactants (Schenk et al. 2021).

### Intra-specific trait variability

Consistent with previous studies, we observed a high degree of intra-population variability in xylem safety, which is unlikely to be caused by small-scale soil moisture variation and/or competition between neighbouring trees. European beech is known for a high degree of intra-population genetic variability (Wortemann et al. 2011; Aranda et al. 2015; Hajek et al. 2016) and a high degree of within-crown plasticity of *P*_50_ has been reported for European beech (Cochard et al. 1999; Lemoine et al. 2002; Herbette et al. 2010; Schuldt et al. 2016). We speculate that differences in light intensity and vapour pressure deficit within the crown of single trees experienced by given branches during their lifetime, as well as the observed correlation between branch age and *P*_50_, can well explain this heterogeneity. In field studies, it is hardly possible to control for these potentially influential factors. In our study, only similar-sized branches with a comparable number of lateral twigs (mean ± SE: 6.3 ± 0.1 n) were sampled in the 30 stands. However, we observed neither a relationship between branch age and branch diameter nor the degree of branch tapering (difference between basipetal and acropetal segment diameter). This made it impossible to exclude older branches during sampling. Furthermore, the growth conditions during the lifespan of branches are difficult to compare. The only option to reduce the high degree of intra-tree variability in embolism resistance is to sample a large number of replicates from each tree, which reduces the number of stands that can be investigated and results in an additional nesting level and corresponding issues with pseudoreplication. Our study with 300 climbed trees on 30 sites reached the limits of field studies in terms of labour effort. Future studies on within-crown variation, within-population variability, and ontogenetic change in *P*_50_ would help to assess the results of provenance and environmental gradient studies of embolism resistance in a broader context.

Besides the high intra-population variability, we also observed considerable between-population variability that was not explained by the fixed effects. For *P*_50_, 29% of the variance resulted from random differences between sites, which might be attributed to unobserved environmental variables, small-scale climatic deviations from long-term trends, or phylogenetic differences between populations. As we included a broad range of climatic and edaphic variables, it is unlikely that all of the unexplained between-population variance is driven by unobserved environmental variables. However, the observed rapid response of beech vessel properties to extreme climate conditions (Zimmermann et al. 2021) suggests that small-scale climate fluctuations may precipitate transient changes in embolism resistance at some sites. In either case, the magnitude of the unexplained between-population variability is indicative of a possible phylogenetic signal in embolism resistance, and may thus indicate a potential for the selection of more drought-resistant genotypes.

### Trade-off between xylem safety and efficiency

Previous studies failed to detect any relationship between xylem safety and efficiency in European beech (Cochard et al. 1999; Hajek et al. 2016; Schuldt et al. 2016). In accordance with these results, we did not find a relationship between *P*_50_ and *K*_s_ at the intra-specific level (see Fig. S3). At the inter-specific level, however, this trade-off has repeatedly been reported (e.g., Wheeler et al. 2005; Maherali et al. 2006; Schumann et al. 2019; van der Sande et al. 2019). It is not yet understood why intra-specific studies commonly fail to detect this anticipated relationship (cf. Tyree et al. 1994), while inter-specific studies often confirm it. One may speculate that due to the restricted range in vessel diameter variation and embolism resistance found in intra-specific studies, they often lack the power to detect such a relation. Furthermore, vessel diameter is only indirectly related to embolism resistance through its relatedness to inter-vessel pit membrane thickness (Isasa et al. unpublished). As shown by a modelling approach, this ultra-structural wood trait, in combination with the number of pits per vessel, is directly affecting xylem embolism resistance (Kaack et al. 2021). In either case, when comparing relationships at the within- and between-species level, there is a risk of succumbing to ecological fallacies, i.e., using conclusions from between-group relationships to draw inferences on within-group relationships or vice versa. The driving forces for differences on the two levels of aggregation are likely very different, with between-species differences being driven by natural selection for trait combinations associated with different drought resistance strategies, and within-species differences to a large degree mirroring ontogenetic changes that often reflect relatively static allometric scaling rules. Accordingly, the existence of an inter-specific trade-off thus does not necessarily imply the existence of a within-species relationship.

## Conclusion

To our knowledge, this study provides the largest intra-specific dataset on the xylem safety and efficiency of a tree species across a climatic gradient available so far. European beech, the most important tree species of Central Europe’s natural forest vegetation and an important timber species, seems to be vulnerable to climate warming-related drought and heat events. This became widely evident during the extreme 2018/19 drought event, when exceptionally low foliar water potentials crossing the threshold for xylem hydraulic failure were observed at various sites in Central Europe (Schuldt et al. 2020; Walthert et al. 2021). Understanding to what extent European beech is capable of acclimating its branch hydraulic system to achieve greater drought tolerance is therefore of high importance.

In our gradient study, *P*_50_ differed by 1 MPa across the sites, but this intra-specific trait variability was neither related to changes in the climatic water balance nor to tree height or neighbourhood composition. Instead, soil water availability as approximated by the soil capacity for plant-available water was the most important determinant, even though the explained variance was small. The expected increase in embolism resistance towards drier sites as a result of adaptation or acclimation to drought could therefore only partly be confirmed. Branch age was, however, the by far most important single factor influencing xylem embolism resistance, which is unrelated to any site or stand attribute. A much greater proportion of variance in *P*_50_ is attributable to phenotypic or genotypic variation between tree individuals and assumable to variation across the crown. Certainly, the considerable hydraulic plasticity within European beech populations may represent a solid basis for selection processes. However, the weak influence of water availability on xylem safety indicates that the relevant xylem anatomical traits may be more controlled by factors other than drought, and xylem safety might not be a promising factor for identifying more drought-resistant provenances.

## Supplementary Information

Below is the link to the electronic supplementary material.

Supplementary file1 (PDF 1422 KB)

## Data Availability

All data used in this manuscript are present in the manuscript and its supporting information.

## References

[CR1] Adams HD, Zeppel MJ, Anderegg WR, Hartmann H, Landhäusser SM, Tissue DT, Huxman TE, Hudson PJ, Franz TE, Allen CD, Anderegg LD, Barron-Gafford GA, Beerling DJ, Breshears DD, Brodribb TJ, Bugmann H, Cobb RC, Collins AD, Dickman LT, Duan H, Ewers BE, Galiano L, Galvez DA, Garcia-Forner N, Gaylord ML, Germino MJ, Gessler A, Hacke UG, Hakamada R, Hector A, Jenkins MW, Kane JM, Kolb TE, Law DJ, Lewis JD, Limousin J-M, Love DM, Macalady AK, Martínez-Vilalta J, Mencuccini M, Mitchell PJ, Muss JD, O’Brien MJ, O’Grady AP, Pangle RE, Pinkard EA, Piper FI, Plaut JA, Pockman WT, Quirk J, Reinhardt K, Ripullone F, Ryan MG, Sala A, Sevanto S, Sperry JS, Vargas R, Vennetier M, Way DA, Xu C, Yepez EA, McDowell NG (2017). A multi-species synthesis of physiological mechanisms in drought-induced tree mortality. Nat Ecol Evol.

[CR2] Allen CD, Macalady AK, Chenchouni H, Bachelet D, McDowell N, Vennetier M, Kitzberger T, Rigling A, Breshears DD, Hogg EH, Gonzalez P, Fensham R, Zhang Z, Castro J, Demidova N, Lim J-H, Allard G, Running SW, Semerci A, Cobb N (2010). A global overview of drought and heat-induced tree mortality reveals emerging climate change risks for forests. For Ecol Manag.

[CR3] Allen CD, Breshears DD, McDowell NG (2015). On underestimation of global vulnerability to tree mortality and forest die-off from hotter drought in the Anthropocene. Ecosphere.

[CR4] Ambrose AR, Sillett SC, Dawson TE (2009). Effects of tree height on branch hydraulics, leaf structure and gas exchange in California redwoods. Plant Cell Environ.

[CR5] Ambrose AR, Baxter WL, Wong CS, Burgess SS, Williams CB, Næsborg RR, Koch GW, Dawson TE (2016). Hydraulic constraints modify optimal photosynthetic profiles in giant sequoia trees. Oecologia.

[CR6] Anderegg WR, Klein T, Bartlett M, Sack L, Pellegrini AF, Choat B, Jansen S (2016). Meta-analysis reveals that hydraulic traits explain cross-species patterns of drought-induced tree mortality across the globe. PNAS.

[CR7] Anfodillo T, Petit G, Crivellaro A (2013). Axial conduit widening in woody species: a still neglected anatomical pattern. IAWA J.

[CR8] Aranda I, Cano FJ, Gascó A, Cochard H, Nardini A, Mancha JA, López R, Sánchez-Gómez D (2015). Variation in photosynthetic performance and hydraulic architecture across European beech (*Fagus sylvatica* L.) populations supports the case for local adaptation to water stress. Tree Physiol.

[CR9] Aranda I, Bahamonde HA, Sánchez-Gómez D (2017). Intra-population variability in the drought response of a beech (*Fagus sylvatica* L.) population in the southwest of Europe. Tree Physiol.

[CR10] Awad H, Barigah T, Badel E, Cochard H, Herbette S (2010). Poplar vulnerability to xylem cavitation acclimates to drier soil conditions. Physiol Plant.

[CR11] Bartoń K (2020) MuMIn: multi-model inference. MuMIn v. 1.43.17: 2020-04-14. https://CRAN.R-project.org/package=MuMIn

[CR12] Bates D, Mächler M, Bolker B, Walker S (2015). Fitting linear mixed-effects models using lme4. J Stat Softw.

[CR13] Baudis M, Ellerbrock RH, Felsmann K, Gessler A, Gimbel K, Kayler Z, Puhlmann H, Ulrich A, Weiler M, Welk E, Bruelheide H (2014). Intraspecific differences in responses to rainshelter-induced drought and competition of *Fagus sylvatica* L. across Germany. For Ecol Manag.

[CR14] Beikircher B, Mayr S (2009). Intraspecific differences in drought tolerance and acclimation in hydraulics of *Ligustrum vulgare* and *Viburnum lantana*. Tree Physiol.

[CR15] Berzaghi F, Wright IJ, Kramer K, Oddou-Muratorio S, Bohn FJ, Reyer CP, Sabaté S, Sanders TG, Hartig F (2020). Towards a new generation of trait-flexible vegetation models. Trends Ecol Evol.

[CR16] Bittencourt PR, Oliveira RS, da Costa AC, Giles AL, Coughlin I, Costa PB, Bartholomew DC, Ferreira LV, Vasconcelos SS, Barros FV, Junior JA, Oliveira AA, Mencuccini M, Meir P, Rowland L (2020). Amazonia trees have limited capacity to acclimate plant hydraulic properties in response to long-term drought. Glob Change Biol.

[CR17] Boessenkool B (2019) rdwd: select and download climate data from ‘DWD’ (German Weather Service). rdwd v. 1.2.0: 2019-10-26. https://www.CRAN.R-project.org/package=rdwd

[CR18] Bolte A, Czajkowski T, Kompa T (2007). The north-eastern distribution range of European beech a review. Forestry.

[CR19] Braun S, de Witte LC, Hopf SE (2020). Auswirkungen des Trockensommers 2018 auf Flächen der Interkantonalen Walddauerbeobachtung. Schweiz Z Forstwes.

[CR20] Bresson CC, Vitasse Y, Kremer A, Delzon S (2011). To what extent is altitudinal variation of functional traits driven by genetic adaptation in European oak and beech?. Tree Physiol.

[CR21] Brodribb TJ, Powers J, Cochard H, Choat B (2020). Hanging by a thread? Forests and drought. Science.

[CR22] Buiteveld J, Vendramin GG, Leonardi S, Kramer K, Geburek T (2007). Genetic diversity and differentiation in European beech (*Fagus sylvatica* L.) stands differing in management history. For Ecol Manag.

[CR23] Burgess SS, Pittermann J, Dawson TE (2006). Hydraulic efficiency and safety of branch xylem increases with height in *Sequoia sempervirens* (D. Don) crowns. Plant Cell Environ.

[CR24] Carminati A, Javaux M (2020). Soil rather than xylem vulnerability controls stomatal response to drought. Trends Plant Sci.

[CR25] Carsjens C, Ngoc QN, Guzy J, Knutzen F, Meier IC, Müller M, Finkeldey R, Leuschner C, Polle A (2014). Intra-specific variations in expression of stress-related genes in beech progenies are stronger than drought-induced responses. Tree Physiol.

[CR26] Choat B, Sack L, Holbrook NM (2007). Diversity of hydraulic traits in nine *Cordia* species growing in tropical forests with contrasting precipitation. New Phytol.

[CR27] Choat B, Brodribb TJ, Brodersen CR, Duursma RA, López R, Medlyn BE (2018). Triggers of tree mortality under drought. Nature.

[CR28] Christensen-Dalsgaard KK, Tyree MT (2014). Frost fatigue and spring recovery of xylem vessels in three diffuse-porous trees in situ. Plant Cell Environ.

[CR29] Cochard H, Lemoine D, Dreyer E (1999). The effects of acclimation to sunlight on the xylem vulnerability to embolism in *Fagus sylvatica* L. Plant Cell Environ.

[CR30] Cochard H, Damour G, Bodet C, Tharwat I, Poirier M, Améglio T (2005). Evaluation of a new centrifuge technique for rapid generation of xylem vulnerability curves. Physiol Plant.

[CR31] Correia DLP, Bouchard M, Filotas É, Raulier F (2019). Disentangling the effect of drought on stand mortality and productivity in northern temperate and boreal forests. J Appl Ecol.

[CR32] Dai Y, Wang L, Wan X (2020). Frost fatigue and its spring recovery of xylem conduits in ring-porous, diffuse-porous, and coniferous species in situ. Plant Physiol Biochem.

[CR33] Das A, Battles J, Stephenson NL, van Mantgem PJ (2011). The contribution of competition to tree mortality in old-growth coniferous forests. For Ecol Manag.

[CR34] Dormann CF, Elith J, Bacher S, Buchmann C, Carl G, Carré G, Marquéz JR, Gruber B, Lafourcade B, Leitão PJ, Münkemüller T, McClean C, Osborne PE, Reineking B, Schröder B, Skidmore AK, Zurell D, Lautenbach S (2013). Collinearity: a review of methods to deal with it and a simulation study evaluating their performance. Ecography.

[CR35] Fajardo A, Martínez-Pérez C, Cervantes-Alcayde MA, Olson ME (2020). Stem length, not climate, controls vessel diameter in two trees species across a sharp precipitation gradient. New Phytol.

[CR36] Fuchs S, Leuschner C, Link RM, Schuldt B (2021). Hydraulic variability of three temperate broadleaf tree species along a water availability gradient in Central Europe. New Phytol.

[CR37] Fukuda K, Kawaguchi D, Aihara T, Ogasa MY, Miki NH, Haishi T, Umebayashi T (2015). Vulnerability to cavitation differs between current-year and older xylem: non-destructive observation with a compact magnetic resonance imaging system of two deciduous diffuse-porous species. Plant Cell Environ.

[CR38] Geßler A, Keitel C, Kreuzwieser J, Matyssek R, Seiler W, Rennenberg H (2006). Potential risks for European beech (*Fagus sylvatica* L.) in a changing climate. Trees.

[CR39] Gleason SM, Westoby M, Jansen S, Choat B, Hacke UG, Pratt RB, Bhaskar R, Brodribb TJ, Bucci SJ, Cao K-F, Cochard H, Delzon S, Domec J-C, Fan Z-X, Feild TS, Jacobsen AL, Johnson DM, Lens F, Maherali H, Martínez-Vilalta J, Mayr S, McCulloh KA, Mencuccini M, Mitchell PJ, Morris H, Nardini A, Pittermann J, Plavcová L, Schreiber SG, Sperry JS, Wright IJ, Zanne AE (2016). Weak tradeoff between xylem safety and xylem-specific hydraulic efficiency across the world’s woody plant species. New Phytol.

[CR40] González-Muñoz N, Sterck F, Torres-Ruiz JM, Petit G, Cochard H, von Arx G, Lintunen A, Caldeira MC, Capdeville G, Copini P, Gebauer R, Grönlund L, Hölttä T, Lobo-do-Vale R, Peltoniemi M, Stritih A, Urban J, Delzon S (2018). Quantifying in situ phenotypic variability in the hydraulic properties of four tree species across their distribution range in Europe. PLoS ONE.

[CR41] Hacke UG, Sperry JS (2001). Functional and ecological xylem anatomy. Perspect Plant Ecol Evol.

[CR42] Hacke UG, Stiller V, Sperry JS, Pittermann J, McCulloh KA (2001). Cavitation fatigue. Embolism and refilling cycles can weaken the cavitation resistance of xylem. Plant Physiol.

[CR43] Hacke UG, Spicer R, Schreiber SG, Plavcová L (2017). An ecophysiological and developmental perspective on variation in vessel diameter. Plant Cell Environ.

[CR44] Hajek P, Seidel D, Leuschner C (2015). Mechanical abrasion, and not competition for light, is the dominant canopy interaction in a temperate mixed forest. For Ecol Manag.

[CR45] Hajek P, Kurjak D, von Wühlisch G, Delzon S, Schuldt B (2016). Intraspecific variation in wood anatomical, hydraulic, and foliar traits in ten European beech provenances differing in growth yield. Front Plant Sci.

[CR46] Hajek P, Link RM, Nock C, Bauhus J, Gebauer T, Gessler A, Kovach K, Messier C, Paquette A, Saurer M, Scherer-Lorenzen M, Rose L, Schuldt B (2020) Mutually inclusive mechanisms of drought-induced tree mortality. bioRxiv **(2020.12.17.423038)** 10.1111/gcb.16146 35246895

[CR47] Hartmann H, Moura CF, Anderegg WR, Ruehr NK, Salmon Y, Allen CD, Arndt SK, Breshears DD, Davi H, Galbraith D, Ruthrof KX, Wunder J, Adams HD, Bloemen J, Cailleret M, Cobb R, Gessler A, Grams TE, Jansen S, Kautz M, Lloret F, O’Brien M (2018). Research frontiers for improving our understanding of drought-induced tree and forest mortality. New Phytol.

[CR48] Hegyi F, Fries J (1974). A simulation model for managing jack pine stands. Growth models for tree and stand simulation.

[CR49] Herbette S, Wortemann R, Awad H, Huc R, Cochard H, Barigah TS (2010). Insights into xylem vulnerability to cavitation in *Fagus sylvatica* L.: phenotypic and environmental sources of variability. Tree Physiol.

[CR50] Herbette S, Charrier O, Cochard H, Barigah TS (2020). Delayed effect of drought on the xylem vulnerability to embolism in *Fagus sylvatica*. Can J for Res.

[CR51] Hertel D, Strecker T, Müller-Haubold H, Leuschner C (2013). Fine root biomass and dynamics in beech forests across a precipitation gradient—is optimal resource partitioning theory applicable to water-limited mature trees?. J Ecol.

[CR52] Ishii HT, Jennings GM, Sillett SC, Koch GW (2008). Hydrostatic constraints on morphological exploitation of light in tall *Sequoia sempervirens* trees. Oecologia.

[CR53] Kaack L, Weber M, Isasa E, Karimi Z, Li S, Pereira L, Trabi CL, Zhang Y, Schenk HJ, Schuldt B, Schmidt V, Jansen S (2021). Pore constrictions in intervessel pit membranes provide a mechanistic explanation for xylem embolism resistance in angiosperms. New Phytol.

[CR54] Knipfer T, Reyes C, Earles JM, Berry ZC, Johnson DM, Brodersen CR, McElrone AJ (2019). Spatiotemporal coupling of vessel cavitation and discharge of stored xylem water in a tree sapling. Plant Physiol.

[CR55] Koch GW, Sillett SC, Jennings GM, Davis SD (2004). The limits to tree height. Nature.

[CR56] Kuznetsova A, Brockhoff PB, Christensen RHB (2017). lmerTest package: tests in linear mixed effects models. J Stat Softw.

[CR57] Lakatos F, Molnár M (2009) Mass mortality of beech (*Fagus sylvatica* L.) in South-West Hungary. Acta Silv. et Lignaria Hungarica:75–82.

[CR58] Lamy J-B, Delzon S, Bouche PS, Alia R, Vendramin GG, Cochard H, Plomion C (2014). Limited genetic variability and phenotypic plasticity detected for cavitation resistance in a Mediterranean pine. New Phytol.

[CR59] Larter M, Pfautsch S, Domec J-C, Trueba S, Nagalingum N, Delzon S (2017). Aridity drove the evolution of extreme embolism resistance and the radiation of conifer genus Callitris. New Phytol.

[CR60] Lemaire C, Quilichini Y, Brunel-Michac N, Santini J, Berti L, Cartailler J, Conchon P, Badel É, Herbette S (2020). Plasticity of the xylem vulnerability to embolism in poplar relies on quantitative pit properties rather than on pit structure. Tree Physiol.

[CR61] Lemoine D, Cochard H, Granier A (2002). Within crown variation in hydraulic architecture in beech (*Fagus sylvatica* L.): evidence for a stomatal control of xylem embolism. Ann for Sci.

[CR62] Leuschner C (2020). Drought response of European beech (*Fagus sylvatica* L.)—a review. Perspect Plant Ecol Evol.

[CR63] Leuschner C, Ellenberg H (2017). Ecology of Central European forests.

[CR64] Li X, Blackman CJ, Choat B, Duursma RA, Rymer PD, Medlyn BE, Tissue DT (2018). Tree hydraulic traits are coordinated and strongly linked to climate-of-origin across a rainfall gradient. Plant Cell Environ.

[CR65] Li Q, Zhao M, Wang N, Liu S, Wang J, Zhang W, Yang N, Fan P, Wang R, Wang H, Du N (2020). Water use strategies and drought intensity define the relative contributions of hydraulic failure and carbohydrate depletion during seedling mortality. Plant Physiol Biochem.

[CR66] Link RM (2020) corrmorant: flexible correlation matrices based on ‘ggplot2’. corrmorant v. 0.0.0.9007: 2020-04-09. http://www.github.com/r-link/corrmorant

[CR67] Liu H, Gleason SM, Hao G, Hua L, He P, Goldstein G, Ye Q (2019). Hydraulic traits are coordinated with maximum plant height at the global scale. Sci Adv.

[CR68] Lübbe T, Schuldt B, Leuschner C (2017). Acclimation of leaf water status and stem hydraulics to drought and tree neighbourhood: alternative strategies among the saplings of five temperate deciduous tree species. Tree Physiol.

[CR69] Lübbe T, Lamarque LJ, Delzon S (2021). High variation in hydraulic efficiency but not xylem safety between roots and branches in four temperate broad-leaved tree species. Funct Ecol.

[CR70] Maherali H, Pockman WT, Jackson RB (2004). Adaptive variation in the vulnerability of woody plants to xylem cavitation. Ecology.

[CR71] Maherali H, Moura CE, Caldeira MC, Willson CJ, Jackson RB (2006). Functional coordination between leaf gas exchange and vulnerability to xylem cavitation in temperate forest trees. Plant Cell Environ.

[CR72] Martínez-Vilalta J, Cochard H, Mencuccini M, Sterck F, Herrero A, Korhonen JF, Llorens P, Nikinmaa E, Nolè A, Poyatos R, Ripullone F, Sass-Klaassen U, Zweifel R (2009). Hydraulic adjustment of Scots pine across Europe. New Phytol.

[CR73] Martin-StPaul NK, Limousin J-M, Vogt-Schilb H, Rodríguez-Calcerrada J, Rambal S, Longepierre D, Misson L (2013). The temporal response to drought in a Mediterranean evergreen tree: comparing a regional precipitation gradient and a throughfall exclusion experiment. Glob Change Biol.

[CR74] McDowell NG, Adams HD, Bailey JD, Hess M, Kolb TE (2006). Homeostatic maintenance of ponderosa pine gas exchange in response to stand density changes. Ecol Appl.

[CR75] Meier IC, Leuschner C (2008). Genotypic variation and phenotypic plasticity in the drought response of fine roots of European beech. Tree Physiol.

[CR76] Meixner M, Tomasella M, Foerst P, Windt CW (2020). A small-scale MRI scanner and complementary imaging method to visualize and quantify xylem embolism formation. New Phytol.

[CR77] Moreno G, Cubera E (2008). Impact of stand density on water status and leaf gas exchange in *Quercus ilex*. For Ecol Manag.

[CR78] Müller-Haubold H, Hertel D, Seidel D, Knutzen F, Leuschner C (2013). Climate responses of aboveground productivity and allocation in *Fagus sylvatica*: a transect study in mature forests. Ecosystems.

[CR79] Nakagawa S, Johnson PCD, Schielzeth H (2017). The coefficient of determination R^2^ and intra-class correlation coefficient from generalized linear mixed-effects models revisited and expanded. J R Soc Interface.

[CR80] Nguyen QN, Polle A, Pena R (2017). Intraspecific variations in drought response and fitness traits of beech (*Fagus sylvatica* L.) seedlings from three provenances differing in annual precipitation. Trees.

[CR81] Nicotra AB, Atkin OK, Bonser SP, Davidson AM, Finnegan EJ, Mathesius U, Poot P, Purugganan MD, Richards CL, Valladares F, van Kleunen M (2010). Plant phenotypic plasticity in a changing climate. Trends Plant Sci.

[CR82] Noyer E, Lachenbruch B, Dlouhá J, Collet C, Ruelle J, Ningre F, Fournier M (2017). Xylem traits in European beech (*Fagus sylvatica* L.) display a large plasticity in response to canopy release. Ann for Sci.

[CR83] O’Brien MJ, Engelbrecht BM, Joswig J, Pereyra G, Schuldt B, Jansen S, Kattge J, Landhäusser SM, Levick SR, Preisler Y, Väänänen P, Macinnis-Ng C (2017). A synthesis of tree functional traits related to drought-induced mortality in forests across climatic zones. J Appl Ecol.

[CR84] Ogle K, Barber JJ, Willson C, Thompson B (2009). Hierarchical statistical modeling of xylem vulnerability to cavitation. New Phytol.

[CR85] Olson ME (2019). Plant evolutionary ecology in the age of the extended evolutionary synthesis. Integr Comp Biol.

[CR86] Olson ME, Anfodillo T, Rosell JA, Petit G, Crivellaro A, Isnard S, León-Gómez C, Alvarado-Cárdenas LO, Castorena M (2014). Universal hydraulics of the flowering plants: vessel diameter scales with stem length across angiosperm lineages, habits and climates. Ecol Lett.

[CR87] Olson ME, Soriano D, Rosell JA, Anfodillo T, Donoghue MJ, Edwards EJ, León-Gómez C, Dawson T, Camarero Martínez JJ, Castorena M, Echeverría A, Espinosa CI, Fajardo A, Gazol A, Isnard S, Lima RS, Marcati CR, Méndez-Alonzo R (2018). Plant height and hydraulic vulnerability to drought and cold. PNAS.

[CR88] Olson ME, Anfodillo T, Rosell JA, Martínez-Méndez N (2020). Across climates and species, higher vapour pressure deficit is associated with wider vessels for plants of the same height. Plant Cell Environ.

[CR89] Olson ME, Anfodillo T, Gleason SM, McCulloh KA (2021). Tip-to-base xylem conduit widening as an adaptation: causes, consequences, and empirical priorities. New Phytol.

[CR90] Pammenter NW, Vander WC (1998). A mathematical and statistical analysis of the curves illustrating vulnerability of xylem to cavitation. Tree Physiol.

[CR91] Powers JS, Vargas GG, Brodribb TJ, Schwartz NB, Pérez-Aviles D, Smith-Martin CM, Becknell JM, Aureli F, Blanco R, Calderón-Morales E, Calvo-Alvarado JC, Calvo-Obando AJ, Chavarría MM, Carvajal-Vanegas D, Jiménez-Rodríguez CD, Murillo Chacon E, Schaffner CM, Werden LK, Xu X, Medvigy D (2020). A catastrophic tropical drought kills hydraulically vulnerable tree species. Glob Change Biol.

[CR92] R Core Team (2019) R: a language and environment for statistical computing, Vienna, Austria. R v. 3.6.1: 2019-07-05. https://www.R-project.org/

[CR93] Rehschuh R, Mette T, Menzel A, Buras A (2017). Soil properties affect the drought susceptibility of Norway spruce. Dendrochronologia.

[CR94] Renne RR, Schlaepfer DR, Palmquist KA, Bradford JB, Burke IC, Lauenroth WK (2019). Soil and stand structure explain shrub mortality patterns following global change-type drought and extreme precipitation. Ecology.

[CR95] Rennenberg H, Seiler W, Matyssek R, Gessler A, Kreuzwieser J (2004). Die Buche (*Fagus sylvatica* L.)—ein Waldbaum ohne Zukunft im südlichen Mitteleuropa?. Allgemeine Forst Und Jagdzeitung.

[CR96] Rosas T, Mencuccini M, Barba J, Cochard H, Saura-Mas S, Martínez-Vilalta J (2019). Adjustments and coordination of hydraulic, leaf and stem traits along a water availability gradient. New Phytol.

[CR97] Rowland L, da Costa AC, Galbraith DR, Oliveira RS, Binks OJ, Oliveira AA, Pullen AM, Doughty CE, Metcalfe DB, Vasconcelos SS, Ferreira LV, Malhi Y, Grace J, Mencuccini M, Meir P (2015). Death from drought in tropical forests is triggered by hydraulics not carbon starvation. Nature.

[CR98] Ruiz-Benito P, Lines ER, Gómez-Aparicio L, Zavala MA, Coomes DA (2013). Patterns and drivers of tree mortality in iberian forests: climatic effects are modified by competition. PLoS ONE.

[CR99] Ryan MG, Phillips N, Bond BJ (2006). The hydraulic limitation hypothesis revisited. Plant Cell Environ.

[CR100] Schenk HJ, Michaud JM, Mocko K, Espino S, Melendres T, Roth MR, Welti R, Kaack L, Jansen S (2021). Lipids in xylem sap of woody plants across the angiosperm phylogeny. Plant J.

[CR101] Schuldt B, Knutzen F, Delzon S, Jansen S, Müller-Haubold H, Burlett R, Clough Y, Leuschner C (2016). How adaptable is the hydraulic system of European beech in the face of climate change-related precipitation reduction?. The New Phytol.

[CR102] Schuldt B, Buras A, Arend M, Vitasse Y, Beierkuhnlein C, Damm A, Gharun M, Grams TEE, Hauck M, Hajek P, Hartmann H, Hiltbrunner E, Hoch G, Holloway-Phillips M, Körner C, Larysch E, Lübbe T, Nelson DB, Rammig A, Rigling A, Rose L, Ruehr NK, Schumann K, Weiser F, Werner C, Wohlgemuth T, Zang CS, Kahmen A (2020). A first assessment of the impact of the extreme 2018 summer drought on Central European forests. Basic Appl Ecol.

[CR103] Schumann K, Leuschner C, Schuldt B (2019). Xylem hydraulic safety and efficiency in relation to leaf and wood traits in three temperate Acer species differing in habitat preferences. Trees.

[CR104] Skelton RP, Anderegg LD, Diaz J, Kling MM, Papper P, Lamarque LJ, Delzon S, Dawson TE, Ackerly DD (2021). Evolutionary relationships between drought-related traits and climate shape large hydraulic safety margins in western North American oaks. PNAS.

[CR105] Stojnic S, Suchocka M, Benito-Garzón M, Torres-Ruiz JM, Cochard H, Bolte A, Cocozza C, Cvjetkovic B, de Luis M, Martinez-Vilalta J, Ræbild A, Tognetti R, Delzon S (2018). Variation in xylem vulnerability to embolism in European beech from geographically marginal populations. Tree Physiol.

[CR106] Tai X, Mackay DS, Anderegg WR, Sperry JS, Brooks PD (2017). Plant hydraulics improves and topography mediates prediction of aspen mortality in southwestern USA. New Phytol.

[CR107] Tyree MT, Sperry JS (1989). Vulnerability of xylem to cavitation and embolism. Annu Rev Plant Physiol.

[CR108] Tyree MT, Davis SD, Cochard H (1994). Biophysical perspectives of xylem evolution: is there a tradeoff of hydraulic efficiency for vulnerability to dysfunction?. IAWA J.

[CR109] van der Sande MT, Poorter L, Schnitzer SA, Engelbrecht BM, Markesteijn L (2019). The hydraulic efficiency-safety trade-off differs between lianas and trees. Ecology.

[CR110] van Genuchten MT (1980). A closed-form equation for predicting the hydraulic conductivity of unsaturated soils. Soil Sci Soc Am J.

[CR111] van Genuchten MT, Leij FJ, Yates SR (1991). The RETC code for quantifying the hydraulic functions of unsaturated soils.

[CR112] Waite P-A, Schuldt B, Mathias Link R, Breidenbach N, Triadiati T, Hennings N, Saad A, Leuschner C (2019). Soil moisture regime and palm height influence embolism resistance in oil palm. Tree Physiol.

[CR113] Walthert L, Ganthaler A, Mayr S, Saurer M, Waldner P, Walser M, Zweifel R, von Arx G (2021). From the comfort zone to crown dieback: sequence of physiological stress thresholds in mature European beech trees across progressive drought. Sci Total Environ.

[CR114] Weithmann G, Schuldt B, Link RM, Heil D, Hoeber S, John H, Müller-Haubold H, Schüler L-M, Schumann K, Leuschner C (2022). Leaf trait modification in European beech trees in response to climatic and edaphic drought. Plant Biol.

[CR115] Wheeler JK, Sperry JS, Hacke UG, Hoang N (2005). Inter-vessel pitting and cavitation in woody Rosaceae and other vesselled plants: a basis for a safety versus efficiency trade-off in xylem transport. Plant Cell Environ.

[CR116] Wickham H, Averick M, Bryan J, Chang W, McGowan LDA, François R, Grolemund G, Hayes A, Henry L, Hester J, Kuhn M, Pedersen TL, Miller E, Bache SM, Müller K, Ooms J, Robinson D, Seidel DP, Spinu V, Takahashi K, Vaughan D, Wilke C, Woo K, Yutani H (2019). Welcome to the tidyverse. J. Open Source Softw.

[CR117] Woodruff DR, Bond BJ, Meinzer FC (2004). Does turgor limit growth in tall trees?. Plant Cell Environ.

[CR118] Woodruff DR, Meinzer FC, Lachenbruch B (2008). Height-related trends in leaf xylem anatomy and shoot hydraulic characteristics in a tall conifer: safety versus efficiency in water transport. New Phytol.

[CR119] Wortemann R, Herbette S, Barigah TS, Fumanal B, Alia R, Ducousso A, Gomory D, Roeckel-Drevet P, Cochard H (2011). Genotypic variability and phenotypic plasticity of cavitation resistance in *Fagus sylvatica* L. across Europe. Tree Physiol.

[CR120] Young DJ, Stevens JT, Earles JM, Moore J, Ellis A, Jirka AL, Latimer AM (2017). Long-term climate and competition explain forest mortality patterns under extreme drought. Ecol Lett.

[CR121] Zhang W, Feng F, Tyree MT (2018). Seasonality of cavitation and frost fatigue in *Acer mono* Maxim. Plant Cell Environ.

[CR122] Zimmermann J, Link RM, Hauck M, Leuschner C, Schuldt B (2021). 60-year record of stem xylem anatomy and related hydraulic modification under increased summer drought in ring- and diffuse-porous temperate broad-leaved tree species. Trees.

