# Supplementary material for: Soil water availability and branch age explain variability in xylem safety of European beech in Central Europe
Source: Oecologia. 2022 Feb 25;198(3):629–44. doi: 10.1007/s00442-022-05124-9 (PMC8956530; doi:10.1007/s00442-022-05124-9)
Supplement: Supplementary file 1 — Supplementary file1 (PDF 1422 KB) [file 442_2022_5124_MOESM1_ESM.docx]

## *Oecologia* supplementary information

Article title: Soil water availability and branch age explain variability in xylem safety of European beech across 30 sites in Central Europe

Authors: Greta Weithmann, Roman M. Link, Bat-Enerel Banzragch, Laura Würzberg, Christoph Leuschner, Bernhard Schuldt

Article acceptance date: 14.12.2021

DOI: https://doi.org/10.1007/s00442-022-05124-9

Supplementary information

**Table S1:** Soil characteristics and coordinates of the 30 investigated beech (Fagus sylvatica) forests.

**Table S2:** Summary of all major variables explored.

**Table S3:** Results of the linear mixed effects models.

**Figure S1:** Correlation matrix on site level of climatic variables.

**Figure S2:** Correlation matrix on site level (n = 30) of mean *P*_50_ values and mean *K*_s_ values in relationship to climatic variables.

**Figure S3:** Correlation matrix of hydraulic variables in relationship to branch age, competition index and water availability.

**Figure S4:** (a) Branch age at the basipetal end in relationship to branch age at the acropetal end, and (b) of the mean branch age (mean of acropetal and basipetal age) on the difference of the age between the two ends.

**Figure S5:** (a) Linear regressions of mean branch age on mean leaf size or (b) total leaf area of the corresponding branch, and (c) linear regression of tree height on branch age.

**Figure S6:** Box plots visualizing the age of the investigated branch segments of European beech across the 30 stands.

Table S1: Soil characteristics and coordinates of the 30 investigated beech (*Fagus sylvatica*) forests. Soil properties refer to the upper 60 cm of mineral soil. Given are the bulk density (g cm^-3^), ratios of sand, silt and clay (expressed in % of soil mass without stone content), pH value (H_2_O) of mineral soil, carbon/nitrogen ratio of mineral soil (C_org_/N_t_, g g^-1^), and phosphor concentration of mineral soil (P, µg g^‑1^).

Table S2: Summary of all major variables explored. Given are means ± SE per site of the xylem pressures at 12%, 50% and 88% loss of hydraulic conductance (*P*_12_, *P*_50_ and *P*_88_, respectively), the slope at *P*_50_, and specific conductivity (*K*_s_).

Table S3: Standard errors (*SE*), degrees of freedom (*df*) and test statistics (*t-val.*) of linear mixed effects models with fixed effects for climatic water balance (CWB), plant-available water capacity (AWC), tree height, branch age (log-transformed) and the Hegyi competition index (CI), and random intercepts on site level for *P*_12_, *P*_50_, *P*_88_, slope (log-transformed) and *K*_s_ (n = 298, except for *K_s_*: n = 296).

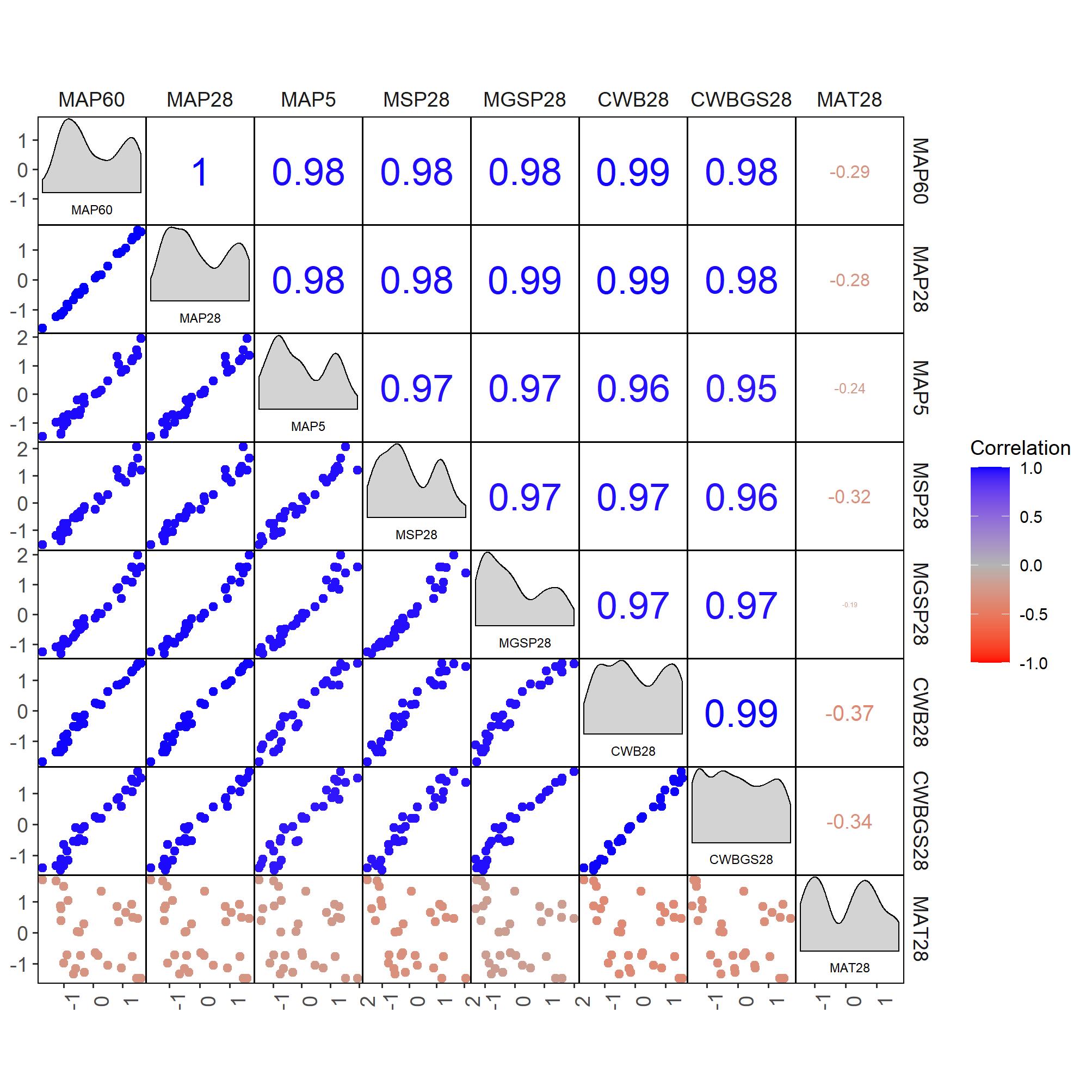


Figure S1: Correlation matrix on site level of climatic variables. Mean annual precipitation for the period 1959 – 2018 (MAP60), for the period 1991 – 2018 (MAP28) and for a time period of 5 years before date of harvest (MAP5), mean early growing season precipitation (April – June, MSP28), mean growing season precipitation (April – September, MGSP28), mean climatic water balance (precipitation – potential evapotranspiration, CWB28), mean growing season climatic water balance (April – September, CWBGS28) and mean annual temperature. Unless otherwise noted means refer to a time period of 1991 – 2018, data were provided by DWD. The lower triangle depicts the variables from x- and y-axis in relation, the upper triangle shows the correlation strength (Pearson’s *r*) and the plot diagonal shows density plots of the corresponding variables.


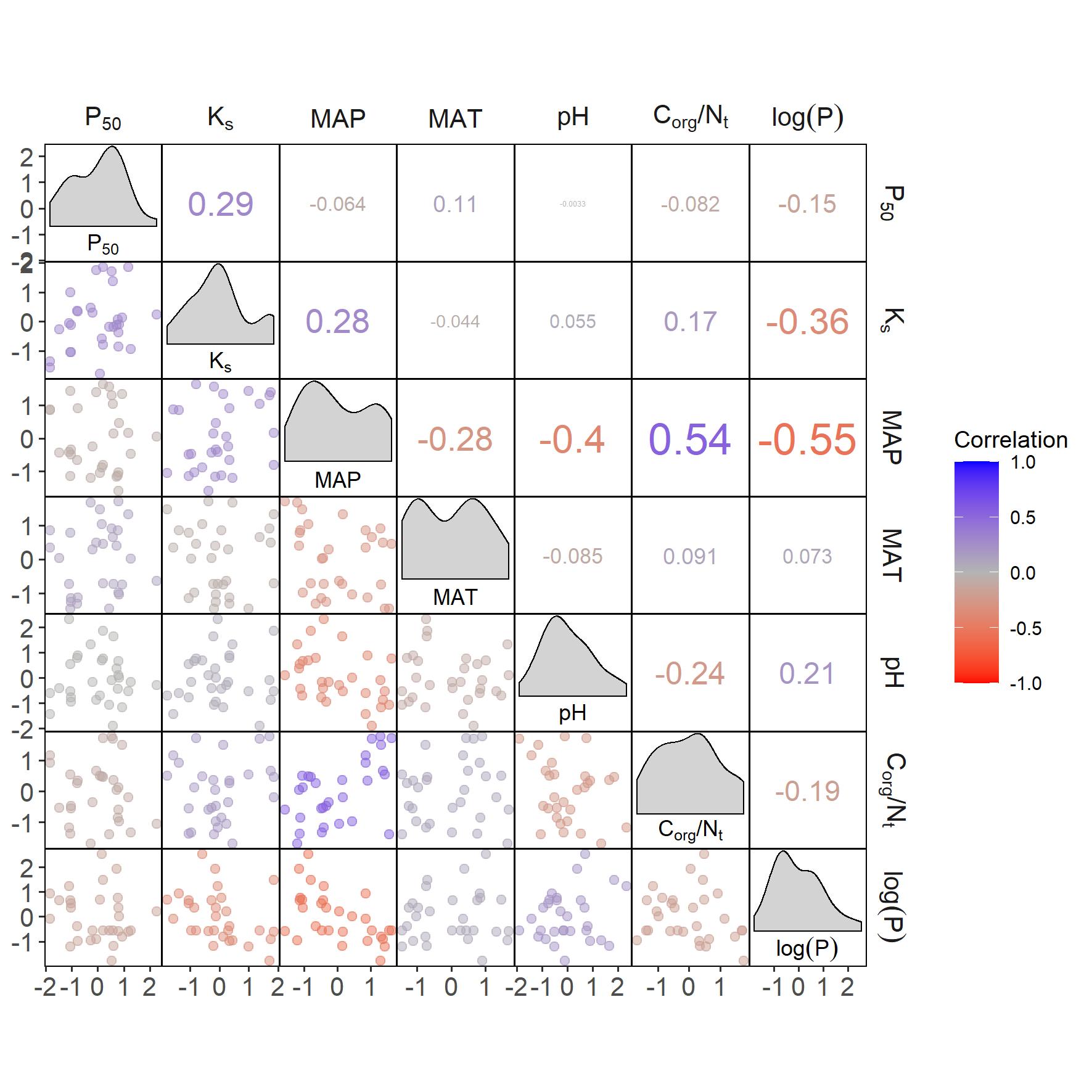


Figure S2: Correlation matrix on site level (n = 30) of mean *P*_50_ values and mean *K*_s_ values of European beech branch segments, climatic variables (mean annual precipitation (MAP, 1991-2018) and mean annual temperature (MAT, 1991 – 2018)) and soil variables (pH value, organic carbon to total nitrogen ratio (C_org_/N_t_), and phosphor content (P, log-transformed)). Values are scaled by the standard deviation and centred around zero. The lower triangle depicts the variables from x- and y-axis in relation, the upper triangle shows the correlation strength (Pearson’s *r*) and the plot diagonal shows density plots of the corresponding variables.


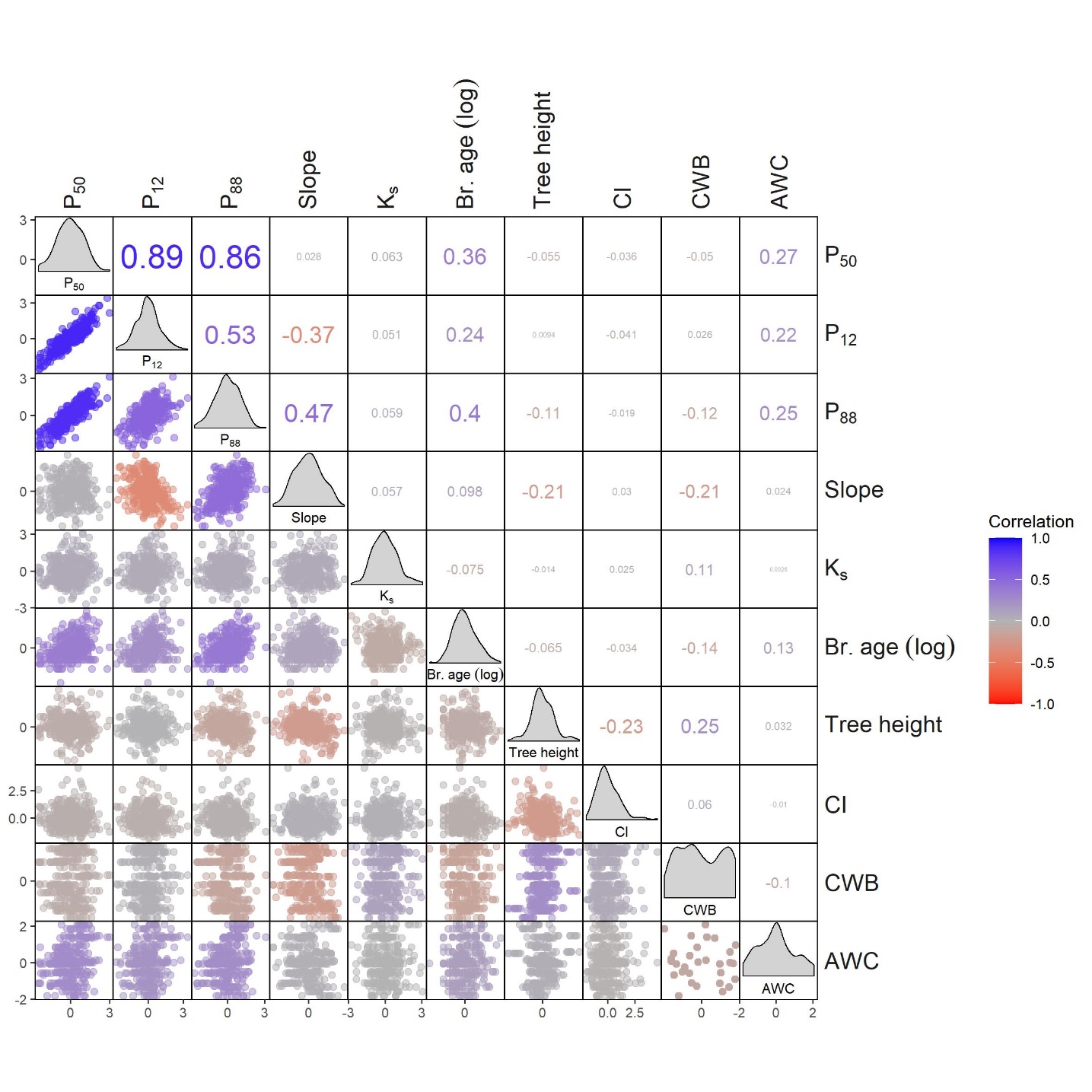


Figure S3: Correlation matrix of *P*_50_-, *P*_12_-, *P*_88_- values, slope of the vulnerability curves, hydraulic conductivity (*K*_s_), age of branch segment (Br. age; log-transformed), tree height, Hegyi competition index (CI), climatic water balance (CWB) and plant-available soil water capacity (AWC). For data on tree level: n = 300, except for CI: n = 298 and for data on site level: n = 30. Values are scaled by the standard deviation and centred around zero. The lower triangle depicts the variables from x- and y-axis in relation, the upper triangle shows the correlation strength (Pearson’s *r*) and the plot diagonal shows density plots of the corresponding variables.


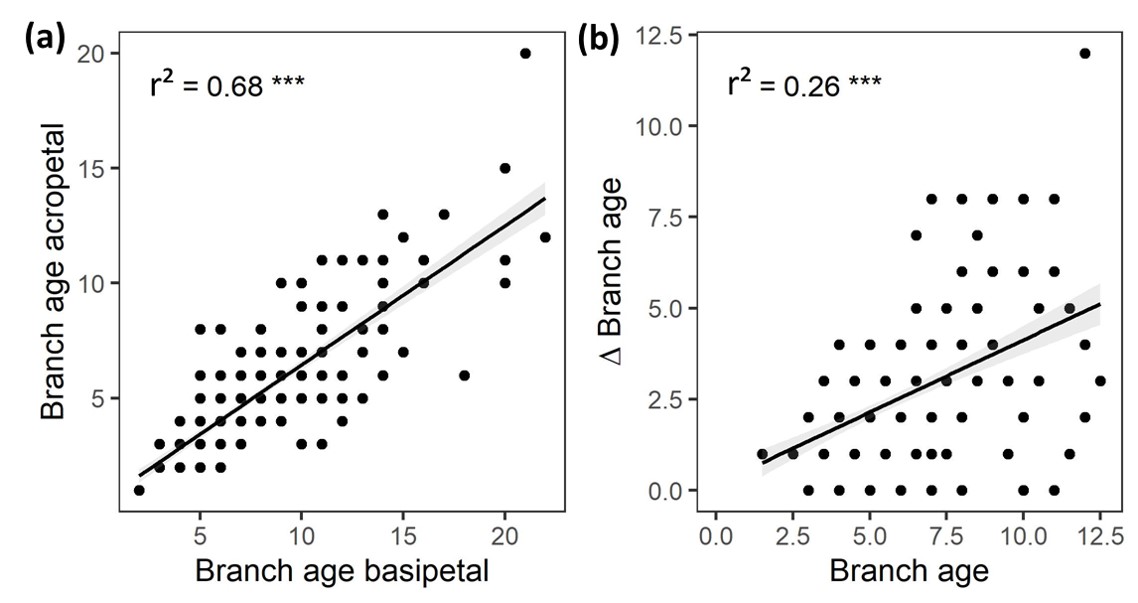


Figure S4: (a) Linear regressions of the branch age (years) at the acropetal end on the branch age of the basipetal end, and (b) of the difference of the age between the two ends on mean branch age (mean of acropetal and basipetal age). The linear regression line with its 95% confidence intervals is shown. Asterisks indicate the level of significance (***: *p* < 0.001).


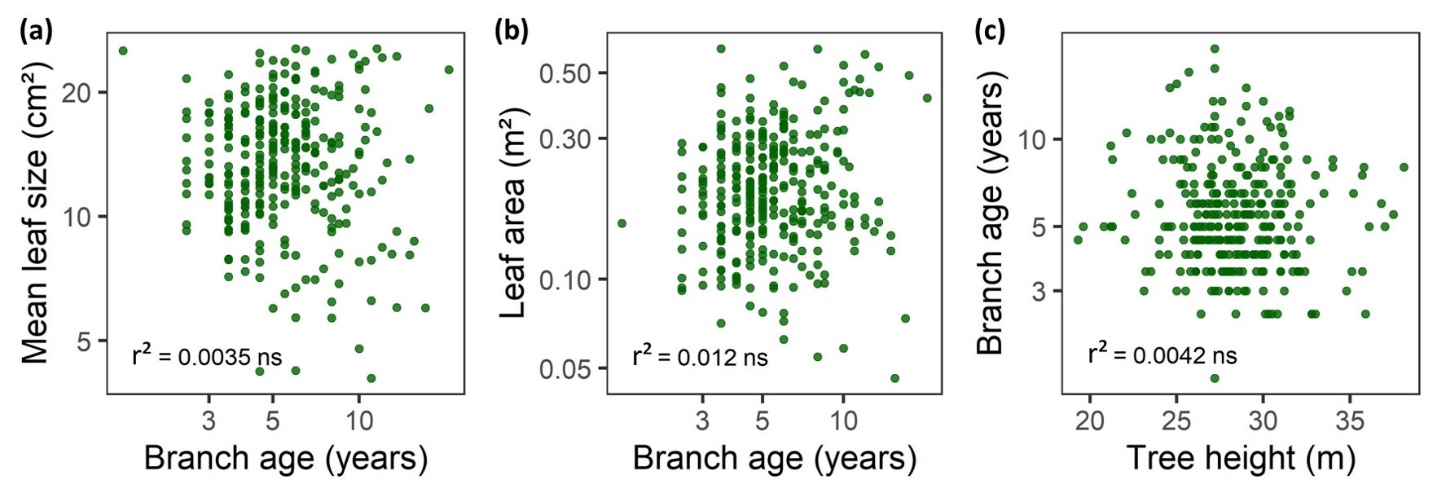


Figure S5: Linear regressions of (a) mean leaf size of the corresponding branch and (b) leaf area of the corresponding branch against mean branch age, and (c) linear regression of branch age on tree height. Given are the explained variance (r²) and level of significance (ns: non-significant relationship).


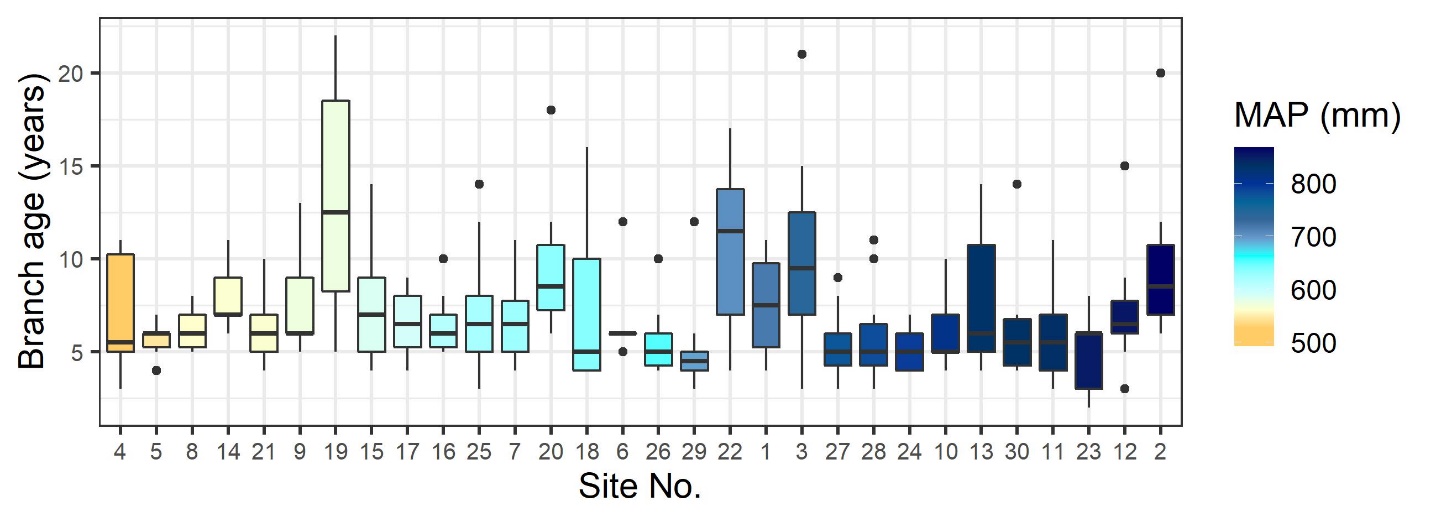


**Figure S6:** Box plots visualizing the age of the investigated branch segments of European beech in the 30 stands (10 trees per stand). Colours indicate mean annual precipitation (MAP, 1991-2018, data provided by DWD) of the different sites (see Figure 1).
